# Obesity and dyslipidemia are associated with partially reversible modifications to DNA hydroxymethylation of apoptosis- and senescence-related genes in swine adipose-derived mesenchymal stem/stromal cells

**DOI:** 10.1186/s13287-023-03372-x

**Published:** 2023-05-25

**Authors:** Logan M. Glasstetter, Tomiwa S. Oderinde, Mohit Mirchandani, Kamalnath Sankaran Rajagopalan, Samer H. Barsom, Roman Thaler, Sarosh Siddiqi, Xiang-Yang Zhu, Hui Tang, Kyra L. Jordan, Ishran M. Saadiq, Andre J. van Wijnen, Alfonso Eirin, Lilach O. Lerman

**Affiliations:** 1 Division of Nephrology and Hypertension, Mayo Clinic, 200 First Street SW, Rochester, MN 55905 USA; 2 Department of Orthopedic Surgery, Mayo Clinic, Rochester, MN USA; 3 Department of Biochemistry, University of Vermont, Burlington, VT USA

**Keywords:** Mesenchymal stem/stromal cells, Epigenetics, Hydroxymethylation, Apoptosis, Senescence, Obesity, Vitamin-C

## Abstract

**Background:**

Obesity dysregulates key biological processes underlying the functional homeostasis, fate decisions, and reparative potential of mesenchymal stem/stromal cells (MSCs). Mechanisms directing obesity-induced phenotypic alterations in MSCs remain unclear, but emerging drivers include dynamic modification of epigenetic marks, like 5-hydroxymethylcytosine (5hmC). We hypothesized that obesity and cardiovascular risk factors induce functionally relevant, locus-specific changes in 5hmC of swine adipose-derived MSCs and evaluated their reversibility using an epigenetic modulator, vitamin-C.

**Methods:**

Female domestic pigs were fed a 16-week Lean or Obese diet (*n* = 6 each). MSCs were harvested from subcutaneous adipose tissue, and 5hmC profiles were examined through hydroxymethylated DNA immunoprecipitation sequencing (hMeDIP-seq) followed by an integrative (hMeDIP and mRNA sequencing) gene set enrichment analysis. For clinical context, we compared 5hmC profiles of adipose tissue-derived human MSCs harvested from patients with obesity and healthy controls.

**Results:**

hMeDIP-seq revealed 467 hyper- (fold change ≥ 1.4; *p*-value ≤ 0.05) and 591 hypo- (fold change ≤ 0.7; *p*-value ≤ 0.05) hydroxymethylated loci in swine Obese- versus Lean-MSCs. Integrative hMeDIP-seq/mRNA-seq analysis identified overlapping dysregulated gene sets and discrete differentially hydroxymethylated loci with functions related to apoptosis, cell proliferation, and senescence. These 5hmC changes were associated with increased senescence in cultured MSCs (p16/CDKN2A immunoreactivity, senescence-associated β-galactosidase [SA-β-Gal] staining), were partly reversed in swine Obese-MSCs treated with vitamin-C, and shared common pathways with 5hmC changes in human Obese-MSCs.

**Conclusions:**

Obesity and dyslipidemia are associated with dysregulated DNA hydroxymethylation of apoptosis- and senescence-related genes in swine and human MSCs, potentially affecting cell vitality and regenerative functions. Vitamin-C may mediate reprogramming of this altered epigenomic landscape, providing a potential strategy to improve the success of autologous MSC transplantation in obese patients.

**Supplementary Information:**

The online version contains supplementary material available at 10.1186/s13287-023-03372-x.

## Introduction

Mesenchymal stem/stromal cells (MSCs) are self-renewing adult somatic stem cells that release trophic factors and extracellular vesicles (EVs) favoring tissue regeneration [1]. The anti-inflammatory, anti-fibrotic, and pro-angiogenic paracrine activities of MSCs, combined with their multilineage differentiation potential, make them an essential component of the endogenous repair system. Moreover, MSCs are candidates for exogenous therapeutic intervention in numerous chronic diseases [1, 2, 3], with the safest contender being autologous cell transplantation. Adipose tissue is an increasingly preferred minimally invasive source of MSC harvest for cellular regenerative therapy. However, in a growing pandemic of obesity [4], fat-derived MSCs can become susceptible to obesity-driven phenotypic alterations that may impair their reparative potency and restrict their autologous therapeutic application in obese patients.

The disease milieu of obesity modifies the microenvironment and endocrine function of adipose tissue, driving chronic inflammation, insulin resistance, and impaired wound healing [5, 6, 7]. We have previously shown that obesity and metabolic syndrome (MetS) instigate functional decline in adipose-derived MSCs, manifesting as skewed differentiation [8], mitochondrial dysfunction [9, 10, 11], impaired immunomodulatory [12] and pro-angiogenic [13, 14] capacity, and increased propensity for senescence [8, 13, 15]. Furthermore, obesity induces broad alterations in the transcriptome and proteome of MSCs [10, 16, 17, 18] and modifies the cargo [19, 20, 21] and morphology [22] of their daughter EVs. Collectively, these obesity-driven changes may blunt the regenerative potency of the afflicted MSCs, thus limiting their therapeutic efficacy [14, 23, 24, 25].

While the mechanisms underlying obesity-induced phenotypic alterations in MSCs remain unclear, a putative driver is epigenetic dysregulation, which results from altered expression or activity of chromatin-modifying enzymes and noncoding RNAs [26]. We have previously shown that obesity and dyslipidemia dysregulate global DNA hydroxymethylation and histone trimethylation levels [27], as well as post-transcriptional modifications involving micro-RNAs [10, 15, 18], in swine MSCs. 5-hydroxymethylcytosine (5hmC) is a stable epigenetic mark [28], derived from 5-methylcytosine (5mC) through action of ten-eleven translocation (TET) methylcytosine dioxygenases [29, 30], that is potentially sensitive to the metabolic and inflammatory derangements in obesity [27, 31, 32, 33]. Moreover, 5hmC modifications are associated with transcriptional regulation [34, 35, 36], may serve as informative biomarkers for cellular state or complex disease status [37, 38, 39], and, importantly, can be favorably reprogrammed *in vitro* by epigenetic modulators, such as vitamin-C (L-ascorbic acid), a cofactor for the TET enzymes [40]. In agreement, we have previously demonstrated reversal of obesity-induced changes in global 5hmC levels of swine MSCs upon co-incubation with vitamin-C [27].

The current study applied hydroxymethylated DNA immunoprecipitation sequencing (hMeDIP-seq) to test the hypothesis that obesity and dyslipidemia induce functionally relevant, locus-specific alterations to DNA hydroxymethylation in swine MSCs that would be reversible with vitamin-C epigenetic reprogramming *in vitro*. Identifying 5hmC changes in genes implicated in salient biological pathways, and testing their reversibility with epigenetic modulators, will assist in the continued development of novel therapeutic strategies to rejuvenate the reparative capacity of MSCs for autologous transplantation in individuals with obesity and cardiovascular risk factors.

## Materials and methods

### Porcine model of experimental obesity

This manuscript adheres to the ARRIVE guidelines for the reporting of animal experiments. Animal studies were approved by the Institutional Animal Care and Use Committee (IACUC). Twelve three-month-old female domestic pigs (Manthei Hog Farm, Elk River, MN) were randomized into two groups (*n* = 6, each): Lean pigs (control) were fed standard chow (13% protein, 2% fat, 6% fiber; Purina Animal Nutrition LLC, MN), while Obese pigs were fed ad libitum with a high-fat/high-fructose diet (5B4L; 16.1% protein, 43.0% ether extract fat, 40.8% carbohydrates; Purina Test Diet, Richmond, IN) [41]. Animals were housed in an accredited facility and provided with free access to water. After 16 weeks of diet, blood samples were obtained, and pigs were euthanized with sodium pentobarbital (100 mg/kg IV, Fatal Plus®, Vortech Pharmaceuticals, Dearborn, MI). 5-10 g of subcutaneous abdominal adipose tissue was collected for MSC isolation.

### Swine systemic measurements

After 16 weeks of diet, swine arterial blood pressure was measured using a pressure transducer in the right carotid artery, and body weight was recorded. Venous levels of total cholesterol, low-density lipoprotein (LDL), and triglycerides were measured by standard procedures. Fasting glucose and insulin levels obtained by standard procedures were used to calculate the homeostasis model assessment of insulin resistance (HOMA-IR) score (fasting plasma glucose × fasting plasma insulin/22.5), as an index of insulin resistance [41].

### Human MSC studies

In human subjects, subcutaneous abdominal fat (0.5-2 g) was collected during bariatric (Obese,* n* = 5) or kidney donation (Lean, *n* = 5) surgeries [13]. Samples were placed on ice and processed for MSC harvesting, expansion, and hMeDIP-seq analysis. Inclusion criteria for the Obese patients were age of 18–80 years and body mass index (BMI) > 30 kg/m^2^. For age- and sex-matched Lean controls, inclusion criteria were age > 18 years, BMI < 30 kg/m^2^, and healthy overall state. For both groups, exclusion criteria were recent stroke or myocardial infarction, solid organ transplant recipients, chronic inflammatory disease, immunosuppressive treatment, pregnancy, active malignancy, or blood thinners/chronic anticoagulant therapy. Informed written consent was obtained from subjects after the study was approved by the Institutional Review Board (IRB) of the Mayo Clinic. Relevant laboratory measures, including total cholesterol, triglycerides, LDL, and fasting glucose were assessed by standard procedures from blood samples. Blood pressure was obtained from the medical records.

### MSC isolation, culture, and characterization

Primary swine and human MSCs isolated from subcutaneous abdominal adipose tissue were expanded in culture, as previously described [13, 27, 42]. Briefly, adipose tissue was enzymatically digested using collagenase-H, filtered through a 100 μm cell strainer and centrifuged to pellet cells. Cells were then cultured for three weeks in T-75 cm^2^ flasks (TPP, Cat.#Z707503) placed within a humidified incubator (37 °C/5% CO_2_), using a growth medium consisting of Advanced Minimum Essential Medium (Gibco, Carlsbad, CA, Cat.#12492013) supplemented with 5% platelet lysate (PLTmax, Mill Creek Life Sciences, Rochester, MN) and 2 mM L-glutamine (Gibco, Cat.#25030081). MSCs were passaged at 60–80% confluence using TrypLe (Gibco, Cat.#12604013), and assays were performed using passages 3–4.

Our previous studies indicate that swine [42] and human [12, 13, 20] MSCs harvested using our lab protocols satisfy the International Society for Cellular Therapy (ISCT) minimum criteria [43]: MSCs are plastic-adherent when maintained in standard culture conditions, express MSC-specific surface markers (CD73/NT5E, CD90/THY1, CD105/ENG) and do not express CD14, CD34, or CD45/PTPRC, and are able to differentiate into osteocytes, chondrocytes, and adipocytes *in vitro*. In the current study, we confirmed expression of MSC-specific markers by imaging flow cytometry (FlowSight, Amnis, Seattle, WA), as previously described [13, 42], and verified tri-lineage differentiation using an MSC Functional Identification Kit (R&D Systems®, Minneapolis, MN, Cat.#SC006) [8, 13].

### hMeDIP-seq analysis

From swine (*n* = 3/group) and human (*n* = 5/group) Lean- and Obese-MSC samples, DNA was extracted using the DNeasy Blood and Tissue Kit (Qiagen, Cat.#69504) with RNase treatment, following the manufacturer’s protocol [44]. DNA concentrations were measured by a spectrophotometer (NanoDrop) and diluted to 100 ng/μL with TE buffer. The aliquot (100μL) of genomic DNA was sonicated using a Bioruptor® Pico instrument (Diagenode) for 7–10 cycles at 30 s on and 30 s off. The size of fragmented DNA was determined using a Fragment Analyzer (Advanced Analytical Technologies, Ankeny, IA) with the High Sensitivity NGS Fragment Analysis Kit (Cat.#DNF-486). Fragmented DNA with an average size of 200 bp was denatured for 10 min at 95 °C, after which 2.5-5 μg of DNA in 1X DNA immunoprecipitation (DIP) buffer (10 mM sodium phosphate, pH 7.0, 140 mM NaCl, 0.05% Triton X-100) was incubated with 1 μg of anti-5hmC antibody from the hybridoma clone EDL HMC 1A (equivalent to Cat.#MABE1093 from MilliporeSigma) for 3 h at 4 °C on a rotator. Dynabeads Protein-G (Thermo-Fisher, Cat.#10003D) were added to the DNA/antibody mixture, and the reaction was incubated at 4 °C overnight on a rotator. Collected beads-antibody-DNA complexes were then thoroughly washed with DIP buffer and TE buffer. The enriched DNA fragments were eluted from the beads, purified using the ssDNA/RNA Clean & Concentrator (Zymo Research, Cat.#D7010), and quantified with the Qubit ssDNA High Sensitivity Assay Kit (Thermo-Fisher Scientific, Cat.#Q10212). DNA libraries were generated from input and enriched DNAs using the Accel-NGS® 1S Plus DNA Library Kit (Swift Biosciences, Cat.#10024), according to the manufacturer’s protocol; they were then sequenced to 51 bp from both ends on an Illumina HiSeq 4000 instrument in the Mayo Clinic Medical Genome Facility.

Bioinformatic analysis of hMeDIP-seq data [42, 44] was performed by aligning paired-end sequenced FASTQ files to the porcine reference genome (susScr 11.1) for swine MSC samples, and to the human reference genome (hg38) for human MSC samples, using Bowtie 2 (v2.3.3.1) [45]. Duplicate reads were removed with MarkDuplicates (PICARD v1.67), and hMeDIP-seq peaks were called using MACS2 [46]. Differential binding analysis of hMeDIP-seq peak data was performed using the DiffBind package (v2.14.0), and the Homer [47] (v4.10) peak annotation tool was used to annotate and assign differential peaks and genomic coverage bins. 5hmC coverage analysis applied per-base coverage of regions of interest, calculated with bedtools (v2.20.0) genomeCoverageBed. Sequence read values for overall exonic 5hmC coverage per gene were calculated as faux-RNA counts [42, 44] using htseq-count [48] (v0.9.1) and then processed by edgeR (v3.28.1). Differentially hydroxymethylated genes in swine Obese- versus Lean-MSCs were presented in heat maps generated using Morpheus (https://software.broadinstitute.org/morpheus/). 5hmC profiles of representative genes (GPX3, MYH7) in swine Obese- and Lean-MSCs were visualized using Integrative Genomics Viewer (IGV) [49].

### mRNA sequencing (mRNA-seq) analysis

mRNA sequencing (mRNA-seq) analysis was performed at the Mayo Clinic Genomic Analysis Core and Bioinformatics Core on samples from swine Obese- and Lean-MSCs (*n* = 3 each), as described [17, 18, 27, 50]. Libraries were prepared (TruSeq RNA Sample Prep Kit v2, Illumina, San Diego, CA) and loaded onto flow cells (8-10 pM) at cluster densities of 700,000/mm^2^, using the standard protocol for the Illumina cBot and cBot Paired-end cluster kit v3. Flow cells were sequenced on an Illumina HiSeq 2000 using TruSeq SBS kit v3 and HCS v2.0.12 data collection software. mRNA-seq data was analyzed using the MAPRSeq v.1.2.1 workflow [51] and the Bioinformatics Core standard tool. Gene expression was normalized to 1 million reads and corrected for gene length (Reads Per Kilobase per Million mapped reads, RPKM) using edgeR 2.6.2 [52]. The mRNA-seq dataset analyzed in this study has been previously published by our group [17].

### Gene sets-based integrative analysis of gene expression and 5hmC profiles of swine MSCs

To investigate the implication of DNA hydroxymethylation on biological and cellular processes altered in Obese- versus Lean-MSCs, we performed a comparative Gene Set Enrichment Analysis (GSEA, version 4.0.3, Broad Institute) [53] on the mRNA-seq and hMeDIP-seq datasets obtained from swine samples. For this purpose, we separately performed GSEA on the mRNA-seq and hMeDIP-seq data files, screening for all online available gene sets (> 31,000) in the Molecular Signatures Database (MSigDB) (https://www.gsea-msigdb.org/gsea/msigdb/index.jsp). The results were ranked by normalized enrichment score (NES) and selected using a threshold of ± 1.4. The filtered gene sets from mRNA-seq and hMeDIP-seq were then cross-compared and visualized with a scatterplot. Genes from gene sets with NES < − 1.4 for both hMeDIP-seq and mRNA-seq were extracted from the MSigDB v7.4 collections, and Gene Ontology: Biological Process (GOBP) overlap was computed, selecting the top 10 overlapping gene sets, given FDR q-value < 0.05. Genes from gene sets with NES > 1.4 for both hMeDIP-seq and mRNA-seq were also extracted and, for genes with a multiplicity of at least 3 in the composite list (i.e., those that appeared in at least 3 of the 95 gene sets), GOBP overlap analysis was performed. The MSigDB GOBP overlap analysis was validated by computing GOTERM_BP_DIRECT Functional Annotation Chart analyses with the Database for Annotation, Visualization and Integrated Discovery (DAVID 2021 Update; https://david-d.ncifcrf.gov/) [54].

### hMeDIP-seq analysis of apoptosis- and senescence-related genes in swine MSCs

To elucidate whether obesity deregulates 5hmC levels in genes related to apoptosis in swine MSCs, we filtered [42] our hMeDIP-seq data by the Gene Ontology (GO) term for apoptotic process (GO:0006915), using gene sets obtained from the MGI database (http://www.informatics.jax.org/genes.shtml) and AmiGo-2 (Organism: *Homo sapiens*; http://amigo.geneontology.org/amigo). For senescence, we filtered genes by the union of two gene sets: Cellular Senescence REACTOME Superpath (https://pathcards.genecards.org/card/cellular_senescence) and the GO term for Cellular Senescence (GO:0090398) from the MGI database. We also filtered the hMeDIP-seq dataset by the GO term for cell population proliferation (GO:0008283), with gene sets obtained from the MGI database and AmiGo-2 (*Homo sapiens*). Apoptosis-, cell population proliferation-, or senescence-associated genes with differential 5hmC levels in Obese- versus Lean-MSCs were then designated based on *p*-value ≤ 0.05 (two-tailed Student's *t*-test) and fold change (Obese-MSCs/Lean-MSCs) ≥ 1.4 (high 5hmC levels) or ≤ 0.7 (low 5hmC levels). Heat maps of differentially hydroxymethylated apoptosis, senescence, and cell population proliferation genes were generated using Morpheus. Differentially hydroxymethylated apoptosis genes were additionally filtered with Venn diagram analysis (Venny 2.1.0) by GO terms for positive regulation of apoptotic process (GO:0043065) and negative regulation of apoptotic process (GO:0043066), using gene sets obtained from the MGI database and AmiGo-2 (*Homo sapiens*). Functional network analysis was performed on differentially hydroxymethylated senescence-related genes using Search Tool for the Retrieval of Interacting Genes (STRING) version 11.5 (http://string-db.org/)) [55]. The minimum required interaction score was set to medium confidence (0.400). For human MSC samples, filtering of the hMeDIP-seq dataset for apoptosis-, cell population proliferation-, and senescence-associated genes was performed, using identical methods to those employed in the pig studies above.

### Protein expression of candidate genes

Expression of several apoptosis-, senescence-, or proliferation-related proteins was quantified in a blinded manner in swine Obese- and Lean-MSCs by Western blotting: DDIT3/CHOP (Cat.#MA1-250, Invitrogen; 1:1000), CASP14 (Cat.#PA5-72903, Invitrogen; 1:1000), MAPK10 (Cat.#17572–1-AP, Proteintech; 1:1000), CDKN2C (Cat.#PA5-78378, Invitrogen; 1:500), and TERT (Cat.#PA5-116024, Invitrogen; 1:500). Results were normalized to the loading control, GAPDH (Cat.#ab8245, abcam; 1:5000).

### Fluorescence microscopy

Fluorescence microscopy studies were performed with a ZEISS Axio Observer-Z1 inverted fluorescence microscope at 20X (LD Plan-Neofluar 20x/0.4 Korr Ph2 M27 objective) or 40X (C-Apochromat 40X-1.20 W Korr objective) magnification, an AxioCamMR3 camera, and ZEN 2 Desk software (ZEISS, Munich, Germany). Images were acquired using excitation/emission filters of 353 nm/465 nm (blue channel), 590 nm/617 nm (red channel), 650 nm/673 nm (far-red channel), and 488 nm/509 nm (green channel). Any adjustment for image presentation purposes was applied to the entire image and not selectively.

### Cell proliferation studies

To study swine MSC proliferation [27], a 24-well plate (TPP, Cat.#Z707791) containing 500µL of growth medium per well was prepared. Each well was seeded with 4.5 × 10^5^ Obese- or Lean-MSCs quantified with a Countess hemocytometer (Thermo-Fisher, NY, USA). The plate was placed in a Cytation™ 5 Cell Imaging Multi-Mode Reader, which also served as an incubator (37 °C/5% CO_2_).

A montage of four phase-contrast images per well was acquired using a wide field of view camera and a 4X objective. Images were taken hourly for 24 h and analyzed using Gen 5 software (version 3.09, Agilent Technologies). Images were preprocessed to enhance contrast by using a dark background (allowing cells to appear white) and a Rolling Ball diameter of 20. These transformed phase-contrast images were then used for cellular analysis. A threshold value of 800, 2 cycles of image smoothing, and background evaluation on 5% of the lowest pixels were found to optimize the identification of cellular structures. A minimum object size of 20 µm was used to exclude cellular debris. The masking effectiveness was visually confirmed by comparing raw phase-contrast images to the transformed and masked images. Confluence was calculated as:

$$\mathrm{Confluence}=\frac{\mathrm{Object \,\,Sum \,\,Area}}{\mathrm{Total\,\, area\,\, of \,\,transformed\,\, phase\text{-}contrast \,\,image}}\times 100\mathrm{\%}$$

In addition, we studied swine Obese- and Lean-MSCs proliferation using immunofluorescent staining of Ki67 (Cat.#ab15580, abcam) and PCNA (Cat.#ab29, abcam); % cellular positivity (normalized for DAPI-stained nuclei) was quantified in a blinded fashion in an average of 10 fields/sample under fluorescence microscopy at 20X magnification. We further performed an MTS assay (CellTiter-96 Aqueous Non-Radioactive Cell Proliferation Assay) in a 96-well plate (30,000 cells/well) and measured absorbance at 490 nm using a BioTek Synergy™ Mx Multi-Mode Reader. Studies were performed in a blinded manner.

### Apoptosis assays

Apoptosis of swine Obese- and Lean-MSCs was detected using the APC Annexin-V/Dead Cell Apoptosis Kit with APC Annexin-V and SYTOX® Green (Invitrogen, Eugene, OR; Cat.#V35113), as described [42]. Analysis was performed using a FlowSight (Amnis, Seattle, WA) flow-cytometer and IDEAS (Amnis, v6.0).

To assay cellular response to challenge, swine Obese- and Lean-MSCs were treated for 24 h *in vitro* with pro-apoptotic staurosporine dissolved in DMSO (Cat.#S6942, Sigma-Aldrich, final concentration 20 nM) [56], or with DMSO alone (0.1% v/v); the final solution was filtered through a 0.22 µm PVDF syringe filter before use. Apoptosis was then evaluated by terminal deoxynucleotidyl transferase dUTP nick-end-labeling (TUNEL) assay using the DeadEnd™ Fluorometric TUNEL System (Promega), with TUNEL-positive cells counted in a blinded fashion in an average of 7 fields/sample under fluorescence microscopy at 40X magnification and normalized to the number of DAPI-stained nuclei.

### Senescence assays

Cellular senescence of swine Obese- versus Lean-MSCs was evaluated by senescence-associated-β-Galactosidase (SA-β-GAL) staining (Cellular Senescence Detection Kit-SPiDER-βGal; Dojindo, Cat.#SG04) according to the vendor’s protocol. At least 20 fields/sample were captured under fluorescence microscopy at 40X magnification; SA-β-GAL + cells were counted and normalized to the number of DAPI-stained nuclei. p16/CDKN2A immunofluorescent staining (Abcam; Cat.#ab118459) was performed following standard protocol, and the percentage of positively stained area was quantified in fluorescence microscopic images (40X magnification) using ZEN® 2012 blue edition (ZEISS, Munich, Germany) [15].

### Epigenetic reprogramming of Obese-MSCs

Swine Obese-MSCs at third passage were cultured for another passage without (Untreated) or with (Treated) co-incubation for 48 h (starting at 80–90% confluence) with vitamin-C (50 μg/mL) [27]; hMeDIP-seq analysis was then performed. Genes with differential 5hmC levels in Treated versus Untreated Obese-MSCs were identified based on *p*-value ≤ 0.05 (two-tailed Student's *t*-test) and 5hmC levels fold change (Treated/Untreated) ≥ 1.4 (high) or ≤ 0.7 (low). Genes with concurrent high 5hmC levels in Obese- versus Lean-MSCs and low 5hmC levels in Treated versus Untreated Obese-MSCs, or vice versa, were identified from scatterplot analysis, filtered for apoptotic process (GO:0006915), cell cycle (GO:0007049; MGI), cell population proliferation (GO:0008283), and cellular senescence (REACTOME Superpath + GO:0090398), and presented in a heat map generated using Morpheus.

### Statistical analysis

GraphPad Prism 9.3.1 (GraphPad Software) and JMP version 14 (SAS Institute, Cary, NC) were used for statistical analysis. Animals were randomly assigned to Lean or Obese groups, and studies were performed in a blinded manner. The normality assumption was tested using the Shapiro–Wilk test. Two-tailed Student’s *t*-test and Mann–Whitney *U* test were used for parametric and non-parametric comparisons, respectively. Data were presented as mean ± standard deviation, except non-normal data, which were displayed as the median and interquartile range (in box-plot format). For cellular proliferation studies, repeated measures two-way ANOVA was performed with Geisser-Greenhouse correction and Šidák's multiple comparisons test, and data were plotted as mean ± standard error of the mean. The apoptosis response to staurosporine was analyzed using the Welch’s *t*-test (due to unequal variances) or with paired *t*-test for paired comparisons. Statistical significance was accepted for *p* ≤ 0.05.

## Results

### Swine systemic characteristics

**Fig. 1:**
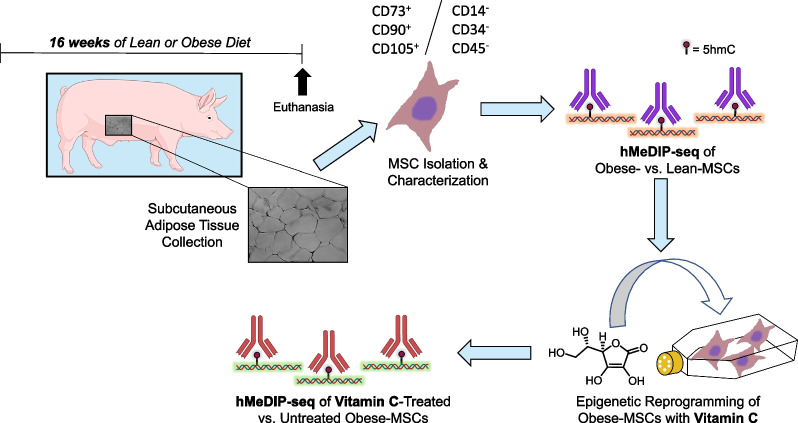
Schematic of experimental design. Three-month-old pigs were fed either standard pig chow (Lean) or a high-fat/high-fructose diet (Obese) for 16 weeks (*n* = 6 each). Mesenchymal stem/stromal cells (MSCs) were isolated from subcutaneous abdominal fat, expanded *in vitro*, and characterized for surface markers. Hydroxymethylated DNA immunoprecipitation sequencing (hMeDIP-seq) was performed on Lean- and Obese-MSCs (*n* = 3 each), and again on Obese-MSCs (*n* = 3) cultured with epigenetic modulator vitamin-C, to determine levels of the 5-hydroxymethylcytosine (5hmC) epigenetic mark. (Illustrations were created with BioRender.com and exported under a paid subscription through the Mayo Clinic)

To explore the effects of diet-induced obesity on the 5hmC profile of MSCs, we used a swine model of experimental obesity previously characterized by our group [41] (Fig. 1). After 16 weeks of diet, Obese as compared with Lean pigs presented with a cluster of cardiovascular risk factors, including hypertension, dyslipidemia (elevated total cholesterol, LDL cholesterol, and triglycerides), and insulin resistance (Table 1). While fasting insulin levels and HOMA-IR score were higher in Obese versus Lean pigs, comparable fasting glucose levels were noted, consistent with development of pre-diabetic MetS.

**Table 1:** Systemic characteristics in experimental swine groups (*n* = 6, each) at 16 weeks

Parameter	Lean	Obese
Body weight (kg)	71.1 ± 13.0	91.1 ± 2.5*
Mean arterial blood pressure (mmHg)	96.4 ± 12.7	127.2 ± 8.5*
Total cholesterol (mg/dL)	81.1 ± 6.9	438.0 ± 81.9*
LDL cholesterol (mg/dL)	32.8 ± 6.0	371.7 ± 143.0*
Triglycerides (mg/dL)	8.0 ± 1.2	19.8 ± 5.8*
Fasting glucose (mg/dL)	127.3 ± 13.7	116.5 ± 17.9
Fasting insulin (µU/mL)	0.4 ± 0.09	0.7 ± 0.04*
HOMA-IR score	0.7 (0.6–0.7)	1.9 (1.6–2.0)*

*LDL* Low-density lipoprotein; *HOMA-IR* Homeostasis model assessment of insulin resistance. Data displayed as mean ± standard deviation, or median (interquartile range)

^*^p < 0.05 vs. Lean

### Obesity/dyslipidemia alters the 5hmC profile of swine MSCs

**Fig. 2:**
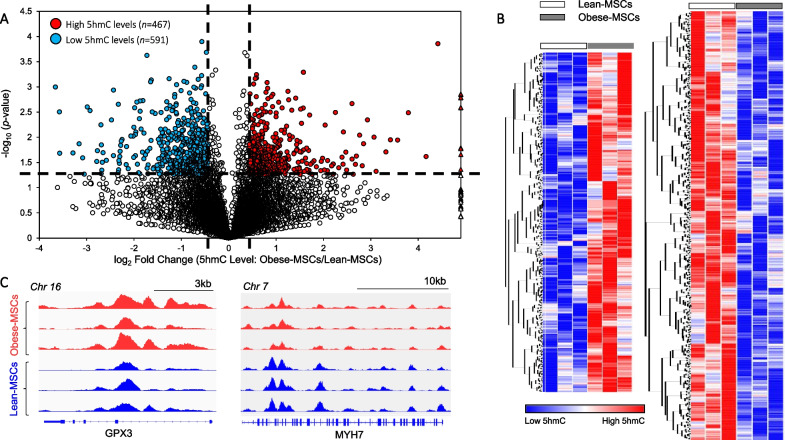
Obesity and dyslipidemia induce epigenetic changes in 5-hydroxymethylcytosine (5hmC) in swine adipose-derived MSCs. **A** Volcano plot of genes with significant changes in overall exonic 5hmC peak levels in Obese-MSCs versus Lean-MSCs, as defined by the following thresholds (dashed lines): *p*-value ≤ 0.05 and fold change (Obese-MSCs/Lean-MSCs) ≥ 1.4 (high 5hmC levels) or ≤ 0.7 (low 5hmC levels). Genes with high (*n* = 467) or low (*n* = 591) 5hmC levels in Obese- versus Lean-MSCs are represented with red or blue circles, respectively, while blank circles indicate genes without significant alterations. Outlier genes with very large fold change values (often due to undetectable 5hmC levels in Lean-MSCs) are displayed as triangles. **B** Heat map of genes with higher (left) or lower (right) 5hmC levels in Obese-MSCs versus Lean-MSCs. **C** Integrative Genomics Viewer (IGV) outputs displaying 5hmC profiles for two representative genes. *n* = 3 per group

MSCs expressed classical markers and possessed multilineage potential, evidenced by their differentiation into osteocytes, chondrocytes, and adipocytes *in vitro* (Additional file 2: Figure S1); as we have previously reported [8], obesity skewed MSC differentiation toward adipocytes and osteocytes. hMeDIP-seq analysis of Obese- and Lean-MSCs identified 5hmC levels in a total of 18,933 genes, of which 467 (2.5%) had high and 591 (3.1%) had low average 5hmC peak levels in Obese-MSCs compared with Lean-MSCs (Fig. 2A–C). Thus, distinctions in the physiological metabolic states of the two experimental swine groups are reflected by general changes in 5hmC epigenetic marks.

### Integrative hMeDIP-/mRNA-seq analysis

**Fig. 3:**
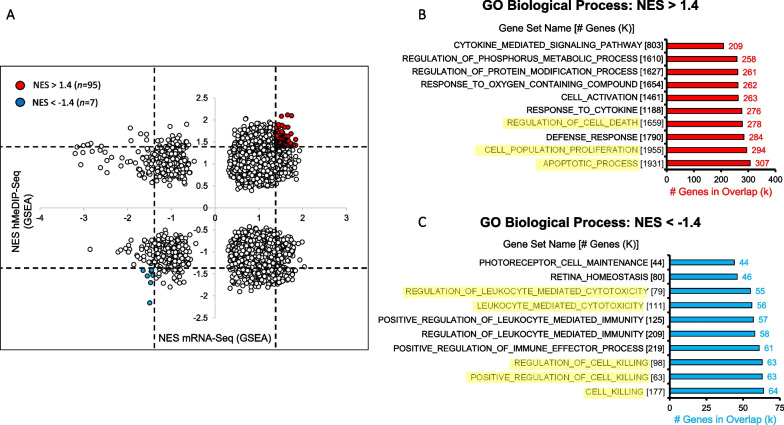
Obesity/dyslipidemia-induced 5hmC marks correlate with changes in gene expression in pathways related to apoptotic process, cell proliferation, and leukocyte-mediated cytotoxicity. **A** Scatterplot of gene sets from the Molecular Signatures Database identifying those with concordant dysregulation of 5hmC levels and mRNA expression in swine Obese- versus Lean-MSCs. For each gene set, the normalized enrichment score (NES) is separately calculated for hMeDIP-seq (vertical axis) and mRNA-seq (horizontal axis) using Gene Set Enrichment Analysis. Based on thresholds (dashed lines) of NES > 1.4 and NES < -1.4, gene sets with higher (*n* = 95) or lower (*n* = 7) levels of both 5hmC and mRNA in Obese- versus Lean-MSCs were marked as red or blue dots, respectively. Genes from the gene sets with **B** NES > 1.4 or **C** NES < -1.4 for both hMeDIP-seq and mRNA-seq were extracted, and classified via Gene Ontology (GO): Biological Process overlap analysis. In panels (**B**, **C**), salient overlapping gene categories are highlighted in yellow

To identify biological processes with concurrent epigenetic and transcriptional derangement in Obese-MSCs compared with Lean-MSCs, we conducted an unbiased, integrative gene set enrichment analysis on mRNA- and hMeDIP-seq data files (Fig. 3A). Given that high levels of 5hmC in gene bodies are generally associated with transcriptional activation [34, 57], we focused on gene sets with the same direction of NES for 5hmC and mRNA. Among members of 95 gene sets upregulated in Obese-MSCs at the level of both mRNA and 5hmC, we identified enrichment for pathways related to apoptotic process (GO:0006915), regulation of cell death (GO:0010941), cell population proliferation (GO:0008283), and response to cytokine (GO:0034097) (Fig. 3B; Additional file 1: Tables S1-S5). The 7 gene sets in Obese-MSCs with downregulated 5hmC and mRNA were enriched for pathways related to cell killing (GO:0001906, GO:003141, GO:0031343) and leukocyte-mediated cytotoxicity (GO:0001909, GO:0001910) (Fig. 3C; Additional file 1: Table S6).

### Changes in 5hmC levels in apoptosis-, proliferation-, and senescence-related genes

**Fig. 4:**
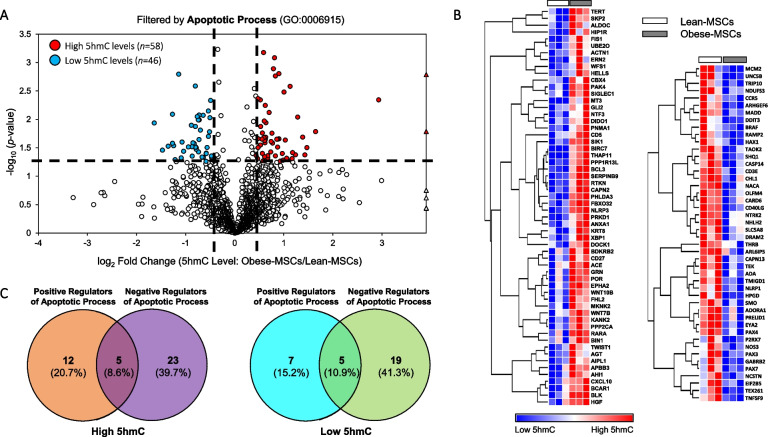
Obesity/dyslipidemia associates with dysregulated 5hmC levels in swine MSCs on genes related to apoptotic process. **A** Volcano plot showing differential 5hmC levels in Obese- versus Lean-MSCs for genes filtered by the Gene Ontology (GO) term for apoptotic process (GO:0006915). For a given gene, differential 5hmC levels entail *p*-value ≤ 0.05 and fold change (Obese-MSCs/Lean-MSCs) ≥ 1.4 (high 5hmC levels) or ≤ 0.7 (low 5hmC levels). Genes with high (*n* = 58) or low (*n* = 46) 5hmC levels in Obese-MSCs versus Lean-MSCs are represented with red or blue markers, respectively. High 5hmC genes with undetectable 5hmC levels in Lean-MSCs are presented as outliers (red triangles). **B** Heat map of genes filtered by apoptotic process (GO:0006915) showing higher (left) or lower (right) 5hmC levels in Obese-MSCs versus Lean-MSCs. **C** Venn diagram analysis shows regulators of apoptotic process among genes with higher (left) or lower (right) 5hmC levels in Obese- versus Lean-MSCs, grouped based on GO terms: positive (GO:0043065) and negative (GO:0043066) regulation of apoptotic process

Because our integrative analysis revealed dysregulation of 5hmC and mRNA in pathways related to apoptosis and cell proliferation (especially their negative regulation; Additional file 1: Tables S1-S5), we subsequently reexamined the genomic distribution of 5hmC in Obese- versus Lean-MSCs, focusing on discrete genes associated with these pathways and cellular senescence (irreversible proliferative arrest). Of 1,847 genes identified in our hMeDIP-seq analysis associated with apoptotic process, 58 (3.1%) had high and 46 (2.5%) had low average 5hmC levels in Obese-MSCs versus Lean-MSCs (Fig. 4A, B), among which negative apoptosis regulators prevailed (Fig. 4C). Two randomly selected candidate apoptosis-related genes with decreased 5hmC levels in Obese-MSCs, DDIT3/CHOP and CASP14, showed correspondingly attenuated protein expression (Additional files 3, 4: Figures S2, S3).

**Fig. 5:**
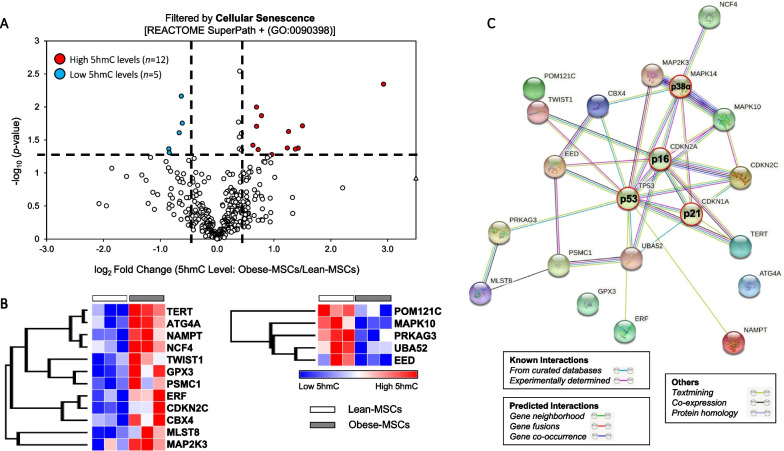
Cellular senescence-associated genes with differential 5hmC levels in swine Obese- versus Lean-MSCs interact richly with canonical markers of senescence. **A** Volcano plot showing differential 5hmC levels in Obese- versus Lean-MSCs for genes filtered by the Cellular Senescence REACTOME Superpath or the GO term for Cellular Senescence (GO:0090398). For a given gene, differential 5hmC levels entail *p*-value ≤ 0.05 and fold change (Obese-MSCs/ Lean-MSCs) ≥ 1.4 (high 5hmC) or ≤ 0.7 (low 5hmC), as shown with dashed lines. Senescence-associated genes with high (*n* = 12) or low (*n* = 5) 5hmC levels in Obese- versus Lean-MSCs are represented with red or blue markers, respectively. **B** Heat map of genes filtered for Cellular Senescence showing higher (left) or lower (right) 5hmC levels in Obese-MSCs versus Lean-MSCs. **C** Functional network analysis of genes with differential 5hmC levels in Obese- versus Lean-MSCs using the STRING database. Network edges indicate both functional and physical protein associations, and color indicates the type of interaction evidence. Inclusion of three classic markers of senescence (p16, p21, and p53), along with stress kinase p38α, in the interaction analysis reveals salient interactions with the epigenetically dysregulated genes

Of 2,020 genes in our hMeDIP-seq analysis associated with cell population proliferation, 54 (2.7%) had high and 52 (2.6%) had low average 5hmC levels in Obese-MSCs (Additional file 5: Figure S4). For the related biological process of cellular senescence, 405 associated genes were identified, of which 12 (3.0%) had high and 5 (1.2%) had low average 5hmC levels in Obese-MSCs (Fig. 5A, B). To interrogate the importance of these genes in a cellular senescence functional network, we examined their interactions with canonical markers of MSC senescence (p16, p21, p53) and with stress kinase p38-α, using the STRING tool, which revealed a rich space of known and predicted interactions (Fig. 5C). Protein expression of candidate cellular senescence- or proliferation-related genes with increased (CDKN2C and TERT) or decreased (MAPK10) 5hmC levels in Obese-MSCs was unaltered (Additional file 3, 4: Figures S2, S3).

### Obesity increases senescence in swine MSCs

**Fig. 6:**
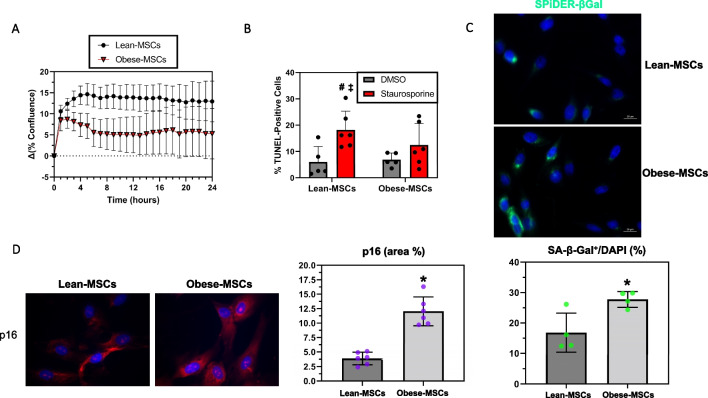
Functional assays indicate increased senescence in swine Obese-MSCs. **A** Cell proliferation, given as change in % confluence across 24 h (mean ± SEM; Obese-MSCs: *n* = 6, Lean-MSCs: *n* = 5) was not significantly different between Obese- and Lean-MSCs. **B** Obese-MSCs, but not Lean-MSCs, showed resistance to induction of apoptosis by staurosporine (dissolved in DMSO; 20 nM for 24 h in vitro; DMSO: *n* = 5 each; staurosporine: *n* = 6 each). **C** Percentage of senescence-associated beta-galactosidase (SA-β-Gal) positively stained cells was increased in Obese- versus Lean-MSCs (*n* = 4 per group). **D** Obese-MSCs also showed increased p16 immunoreactivity (% staining area), compared with Lean-MSCs (*n* = 6 per group). *p-value ≤ 0.05 versus Lean-MSCs; ^**#**^*p*-value ≤ 0.05 versus Lean-MSCs + DMSO; ^**‡**^*p*-value ≤ 0.05 versus Obese-MSCs + DMSO

To investigate the potential functional implications of our epigenetic findings for cell fate decisions, we assayed cellular proliferation and markers of apoptosis or senescence in swine Obese- and Lean-MSCs. No difference in cell proliferation over 24 h was detected by a Cytation™-5 Cell Imaging Multi-Mode Reader (Lean- versus Obese-MSCs: *p*-value = 0.17; Fig. 6A); for confirmation, we also assayed the markers of cell proliferation PCNA (Additional file 6: Figure S5A) and Ki67 (Additional file 6: Figure S5B), which revealed no significant differences between groups. In contrast, a metabolic activity (MTS) assay suggested decreased proliferation in Obese-MSCs (*p*-value = 0.0087; Additional file 6: Figure S5C).

While no differences in basal cellular apoptosis rates (*p*-value = 0.13, Additional files 7, 8: Figures S6, S7A) were detected between Obese- and Lean-MSCs, Obese-MSCs had higher rates of live cells and lower rates of dead cells (both *p*-value = 0.0022; Additional files 7, 8: Figures S6, S7B,C). Nevertheless, Obese-MSCs exhibited resistance to the induction of apoptosis with staurosporine (*p*-value = 0.16 versus DMSO), unlike Lean-MSCs (*p*-value = 0.012 versus DMSO) (Fig. 6B, Additional files 9, 10: S8, S9). In addition, Obese-MSCs showed significantly increased SA-β-Gal positivity (*p*-value = 0.020; Fig. 6C) and p16 immunoreactivity (*p*-value < 0.0001; Fig. 6D) compared with Lean-MSCs, consistent with increased cellular senescence.

### Epigenetic reprogramming partly reverses obesity-induced 5hmC marks in swine Obese-MSCs

**Fig. 7:**
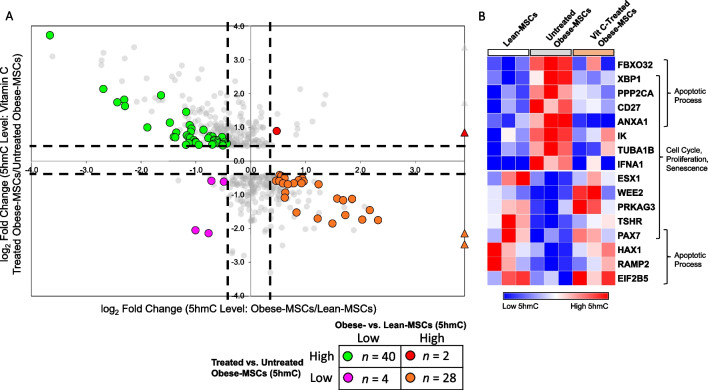
Epigenetic reprogramming of swine MSCs with vitamin-C partly reverses obesity/dyslipidemia-induced changes in hydroxymethylation of genes related to apoptosis and cell proliferation/senescence. **A** Scatterplot of genes in which vitamin-C induced differential 5hmC levels in Obese-MSCs. Genes showing differential 5hmC levels both in Obese- versus Lean-MSCs (x-axis) and in vitamin-C-treated Obese-MSCs versus untreated Obese-MSCs (y-axis) are colored. Differential 5hmC levels entail *p*-value ≤ 0.05 and fold change ≥ 1.4 (high 5hmC levels) or ≤ 0.7 (low 5hmC levels). Outliers (with very large fold change values) are represented with triangle markers. Green points (*n* = 40) represent genes with low 5hmC levels in Obese- versus Lean-MSCs, which are reversed upon vitamin-C treatment, while orange points (*n* = 28) represent genes with high 5hmC levels in Obese- versus Lean-MSCs, which are reversed upon vitamin-C treatment. **B** Heat map of genes for which vitamin-C treatment reverses the differential 5hmC levels observed in Obese-MSCs (orange and green clusters from panel A), filtered for apoptotic process (GO:0006915), cell cycle (GO:0007049), cell population proliferation (GO:0008283), and cellular senescence [REACTOME SuperPath + (GO:0090398)]. *n* = 3 per group

To test whether these altered 5hmC marks may be reversed through epigenetic reprogramming, we co-incubated Obese-MSCs *in vitro* with the epigenetic modulator vitamin-C. For 40 genes with initially low 5hmC levels in Obese- versus Lean-MSCs, vitamin-C increased 5hmC levels in treated versus untreated Obese-MSCs (Fig. 7A). Of these genes, 4 (10.0%) were involved in apoptotic process and 5 (12.5%) in cell population proliferation, cell cycle, and/or cellular senescence (Fig. 7B). For 28 genes with initially high 5hmC levels in Obese-MSCs, vitamin-C decreased 5hmC levels (Fig. 7A). Of these, 5 (17.9%) were annotated for apoptosis and 7 (25%) for cell proliferation, cell cycle, and/or senescence (Fig. 7B). In comparison, vitamin-C treatment of Obese-MSCs further increased 5hmC levels in only 2 genes with initially high 5hmC (versus Lean-MSCs) and decreased 5hmC levels in only 4 genes with initially low 5hmC (Fig. 7A). Hence, vitamin-C modulates 5hmC levels in MSCs and partially reverses the molecular phenotype observed in Obese-MSCs.

### Obesity/dyslipidemia alters 5hmC levels of apoptosis- and senescence-associated genes in human MSCs

**Fig. 8:**
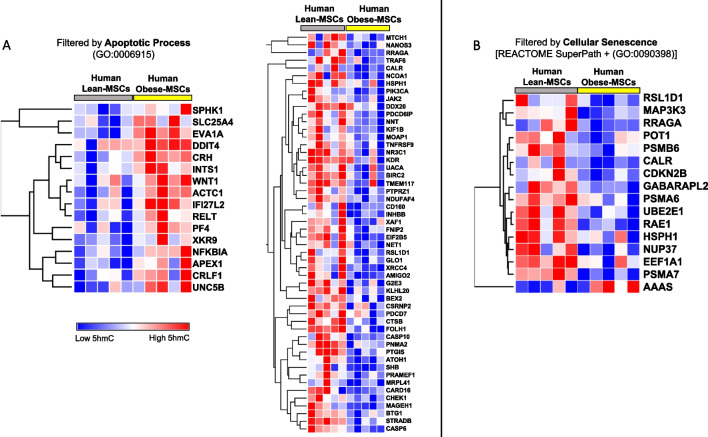
Obesity/dyslipidemia-driven dysregulation of 5hmC epigenetic marks on apoptosis- and senescence-related genes in human adipose-derived MSCs. **A** Heat maps showing high (left; *n* = 16) and low (right; *n* = 52) 5hmC levels in genes filtered by apoptotic process (GO:0006915) in human Obese- versus Lean-MSCs. **B** Heat map showing high (AAAS, *n* = 1) and low (*n* = 15) 5hmC levels in genes filtered by the Cellular Senescence REACTOME Superpath or the GO term for Cellular Senescence (GO:0090398) in human Obese- versus Lean-MSCs. *n* = 5 per group

To assess the clinical relevance of our findings, we conducted a preliminary analysis of the 5hmC profile of MSCs from Obese (*n* = 5) and Lean (*n* = 5) patients. Blood pressure, total cholesterol, LDL, fasting glucose, age, and sex were comparable between Obese and Lean patients, whereas BMI and triglycerides were higher in Obese patients (Additional file 1: Table S7). In hMeDIP-seq analysis, of 1,973 genes associated with apoptotic process, 16 (0.8%) had high and 52 (2.6%) had low average 5hmC levels, in human Obese- versus Lean-MSCs (Fig. 8A, Additional file 11: S10A). Of 420 genes associated with cellular senescence, 15 (3.6%) had low and 1 (0.2%) had high average 5hmC levels (Fig. 8B, Additional file 11: S10B). For cell population proliferation, 2,121 associated genes were identified, of which 45 (2.1%) had low and 27 (1.3%) had high 5hmC levels in human Obese-MSCs (Additional file 12: Figure S11). Thus, results observed for Obese- versus Lean-MSCs in swine translate into similar findings for human MSCs.

## Discussion

This study demonstrates that obesity and dyslipidemia are associated with altered 5hmC levels in apoptosis- and senescence-related genes, with potential functional relevance, in swine adipose tissue-derived MSCs. Our integrative analysis combining high-throughput hMeDIP-seq and mRNA-seq results identified key gene categories with concurrent epigenomic and transcriptomic derangements in the chronic inflammatory milieu of obesity, which underpinned subsequent filtering for genes involved in cell proliferation and fate (apoptosis and senescence). These 5hmC changes were partially reversed in swine MSCs treated with the epigenetic modulator vitamin-C. Moreover, obesity/dyslipidemia-driven epigenetic dysregulation of these pathways was also observed in human MSCs. Therefore, our observations characterize the epigenomic landscape of Obese-MSCs and offer a potential intervention for epigenetic reprogramming of this dysfunctional cellular state.

Obesity is a growing public health concern clinically associated with numerous cardiovascular and metabolic abnormalities. Exposure to this complex disease environment may drive functional deterioration of MSCs residing in perivascular niches of adipose tissue, leading to blunted repair capacity, altered secretome profile, decreased stemness, and propensity for deleterious cell fates, such as senescence or maldifferentiation [8, 13, 14, 22, 23, 24, 58]. These features collectively render Obese-MSCs suboptimal for endogenous repair or autologous therapy.

Reprogramming of cellular memory through epigenetic dysregulation may mediate MSC dysfunction, and therapeutics targeting epigenetic marks may bolster MSC-mediated tissue regeneration [26]. 5hmC is an attractive epigenetic mark for investigation, due to its confined genomic location, biochemical stability, and capacity to be read and interpreted by specific proteins [57]. The 5hmC profile offers an exquisite marker of cell identity and disease; it is sensitive to metabolic perturbations [31, 32], modulates chromatin structure remodeling for active gene expression, and may direct cell fate decisions [57]. We have previously shown that obesity induces widespread global epigenetic alterations at the DNA and histone levels in swine MSCs; importantly, global 5hmC levels correlated with MSC migration and proliferation, and with clinical characteristics of dyslipidemia [27]. Yet, elucidation of the functional role of 5hmC in injured MSCs requires locus-specific methods in a genome-wide analysis [57]. Therefore, we took advantage of high-throughput hMeDIP-seq analysis to detect dynamically modified 5hmC coverage of genes in Obese-MSCs.

We identified 467 hyper- and 591 hypo-hydroxymethylated genomic loci in swine Obese- versus Lean-MSCs. To permit their filtering based on functional relevance, we performed an integrative analysis on genome-wide epigenomic (hMeDIP-seq) and transcriptomic (mRNA-seq) datasets from swine Obese- and Lean-MSCs, and generated GO terms classifying members of overlapping dysregulated gene sets. Gene sets enriched in Obese-MSCs at the level of both 5hmC and mRNA featured genes with functions related to apoptosis, cell proliferation, and regulation of cell death, while those concurrently depleted were linked to leukocyte-mediated cytotoxicity and cell killing.

The functional categories of apoptotic process and cell population proliferation are particularly salient, as inflammation and oxidative stress in obesity may drive MSCs toward cell cycle arrest, senescence, and altered cell death, possibly limiting tissue regeneration [23, 24, 58, 59]. Apoptosis is caspase-mediated programmed cell death that plays an important role in maintaining tissue homeostasis, represents a favorable alternative to pro-inflammatory necrotic cell death, and may be required for the therapeutic function of transplanted MSCs [60]; however, excessive apoptosis could interfere with MSC function and therapy [3]. The contribution of 5hmC modifications to an apoptotic functional signature has been examined in prior work. Progression of alcoholic liver disease was linked to TET-1 inhibition and decreased 5hmC formation that promote apoptotic gene expression in hepatocytes [61]. Furthermore, TET-2 protein-mediated formation of 5hmC has been positively associated with apoptosis and negatively associated with cell cycle progression in human keratinocytes [62]. Finally, alterations in cardiac 5hmC in dilated cardiomyopathy were mapped to genes within pathways related to cell death/apoptosis [63]. With this in mind, we chose to examine apoptotic genes with dysregulated hydroxymethylation in swine Obese-MSCs.

Within the hMeDIP-seq dataset, 12% of genes with high, and 8% with low, 5hmC levels in Obese-MSCs were annotated for the apoptotic process. The distribution of pro- versus anti-apoptotic gene annotations was similar between hyper- and hypo-hydroxymethylated loci, favoring negative regulators of the apoptotic process. Hyper-hydroxymethylated genes in Obese-MSCs included FIS1, which participates in recruitment and activation of procaspase-8 [64], and BCL3, a regulator of NF-κB signaling with cellular pro-survival functions [65]. Hypo-hydroxymethylated genes included DDIT3, which is involved in endoplasmic reticulum stress-induced apoptosis [66], and MADD, a death domain protein that interacts with TNF receptor-1, activates the mitogen-activated protein kinase (MAPK) pathway, and regulates prevention of cell death [67]. Hypo-hydroxymethylation of DDIT3 was accompanied by downregulation of its encoded protein CHOP in Obese-MSCs, consistent with its deactivation in the obese milieu.

Overall, obesity/dyslipidemia did not impose a univocal, directionally consistent impact on 5hmC levels of apoptosis-related genes in swine MSCs, underscoring the dynamic and complex nature of the 5hmC mark. Congruently, no differences in basal apoptosis were detected between swine Obese- and Lean-MSCs, in agreement with our previous observations in human Obese-MSCs [13]. For comparison, apoptosis is either increased [68] or unchanged [69] in adipose-derived MSCs from high-fat diet (HFD)-fed mice, possibly related to differences in the models. Despite unaltered early apoptotic phenotype, rates of cell death were lower for swine Obese-MSCs, in contrast to prior results in adipose-derived MSCs from horses with MetS [70]. Hence, we hypothesized that altered apoptosis phenotype in swine Obese-MSCs may be disclosed during challenge; indeed, staurosporine increased apoptosis in Lean-MSCs but not in Obese-MSCs. This subtle alteration under obesity may account for inconsistent observations made across different conditions.

The obesity-dysregulated cell proliferation signature identified in our integrative 5hmC/mRNA analysis may be related to stem cell exhaustion and aging-related disease [68, 71, 72]. In this context, inflammation, epigenetics, and metabolism converge on cellular senescence, an irreversible proliferative arrest associated with stress response, macromolecular damage, and tissue-destructive secretory features [71, 73]. We have shown that fat inflammation triggers a premature senescence program in Obese-MSCs [8, 13], which may hamper their *in vitro* expansion, blunt their regenerative potency [23, 24], and adversely impact neighboring cells. Removal of senescent fat cells alleviated metabolic dysfunction in obese mice [74], suggesting a feed-forward pathogenic role for cellular senescence in obesity.

While epigenetic dysregulation drives MSC senescence and proliferation loss in aging and metabolic disease [26, 72], few studies have focused on the role of 5hmC marks. Our group recently showed differential hydroxymethylation of senescence-associated loci in MSCs from hypertensive, dyslipidemic swine with kidney dysfunction [42]. Furthermore, genes mapped to sites of high 5hmC and low 5mC in MSCs of advanced-age bone marrow donors showed enrichment for negative regulation of cell cycle [75], whereas therapeutic manipulation of 5hmC and 5mC levels in aged human adipose-derived MSCs increased proliferation and decreased senescence, cell death, and oxidative stress [76].

We identified differentially hydroxymethylated senescence-related genes in swine Obese- versus Lean-MSCs, and assembled them into a protein functional and physical interaction network (STRING) containing three canonical markers of senescent cell cycle arrest, namely cyclin-dependent kinase (CDK) inhibitors p16 (CDKN2A) and p21 (CDKN1A), and tumor-suppressor protein p53 (TP53) [13, 71], as well as MAPK14, a prototypic member of the p38 MAPK family implicated in oxidative stress-induced premature senescence of MSCs [77] and in regulation of the senescence-associated secretory phenotype [78]. The STRING network analysis identified rich protein functional interactions between MAPK14 and two other senescence-associated MAPK genes with high (MAP2K3; a MAPK14 activator) or low (MAPK10) 5hmC in Obese-MSCs. It also identified CDKN2C (p18^INK4C^), a senescence-associated gene and strong inhibitor of stem cell self-renewal [79] with high 5hmC levels in Obese-MSCs, as an interactor of CDKN2A. Finally, our studies revealed network interactions of TERT and TWIST1, both high 5hmC in Obese-MSCs, with CDKN2A and TP53. TERT encodes telomerase reverse transcriptase, which contributes to stem cell self-renewal [72] and is downregulated in senescent adipose tissue-derived MSCs from obese pigs [8] or patients with diabetes [58]. The transcription factor TWIST1 inhibits senescence in human bone marrow-derived MSCs [72].

In parallel with these epigenetic alterations in senescence-related genes, swine Obese-MSCs exhibited increased cellular senescence, as indicated by increased SA-β-Gal positivity [8, 15, 18, 58, 68] and p16 immunoreactivity [13, 15], consistent with previous findings. On the other hand, an irreversible proliferative arrest is a hallmark of full senescence phenotype [73], yet we did not observe significant differences in the proliferative ability of swine Obese- versus Lean-MSCs in vitro, as measured by image-based analysis and levels of proliferation proteins. These findings contrast with prior results in subcutaneous adipose-derived MSCs from patients with obesity [13] and type-2 diabetes [58], but are consistent with previous observations in obese mice [69]. Interestingly, however, we observed in Obese-MSCs blunted metabolic activity that may reflect blunted proliferative ability. Possibly, the lack of advanced co-morbidities (found in human subjects) in our swine model of short-term, diet-induced obesity may account for the partial senescence program observed in Obese-MSCs, which leads to borderline proliferative impairment that unravels in specific conditions or cohorts.

Repatterning of 5hmC in MSCs exposed to obesity might be reversible [26, 32, 71], which may be tested by targeting co-factors of the TET enzymes [27, 33]. Vitamin-C modulates TET-dependent 5hmC formation in embryonic stem cells and fibroblasts, changing gene expression at loci that alter cell fate [40, 80], and it reverses the obesity-prone phenotype of TET1-insufficient HFD mice [81]. We have shown that co-incubation of swine Obese-MSCs with vitamin-C enhances global 5hmC marks [27]. The current study expands our previous observations, demonstrating that obesity-driven dysregulation of 5hmC marks on apoptosis, cell cycle, proliferation, and senescence-related genes in swine Obese-MSCs is partly reversed with *in vitro* vitamin-C treatment, toward levels observed in Lean-MSCs. These results present vitamin-C as a promising epigenetic modulator to potentially restore homeostasis and regenerative potential in dysfunctional MSCs.

For clinical context, we examined the dysregulated 5hmC profile in human Obese-MSCs for genes with functions related to apoptosis, cell proliferation, or senescence. Minimal overlap in differentially hydroxymethylated loci was detected between swine and human Obese-MSCs, encompassing only the apoptosis-related gene EIF2B5 (low 5hmC in Obese-MSCs) and the cell proliferation-related genes IL23R (low 5hmC), FST (high 5hmC), and CALCRL (low 5hmC). Nonetheless, swine and human Obese-MSCs shared common dysregulated pathways. Within the important apoptosis-related caspase pathway, CASP10 and CASP6 had low 5hmC levels in human Obese-MSCs, while CASP14 had low 5hmC levels (as well as decreased protein expression) in swine Obese-MSCs. Furthermore, senescence-associated members of the MAPK pathway had altered 5hmC levels in both swine and human Obese-MSCs. For swine, these genes included the aforementioned MAPK10 and MAP2K3, while for humans, MAP3K3 had low 5hmC levels in Obese-MSCs; MAP3K3 expression is inversely associated with cellular senescence (including levels of p16 and p21) in bone marrow cells [82]. Finally, other important senescence-associated genes such as CDKN2B, which encodes p15^INK4B^, and POT1, which protects against telomere dysfunction and subsequent senescence [83], showed low 5hmC in human Obese-MSCs. These observations imply that obesity alters comparable gene sets in pigs and human subjects.

This study has several limitations. Whether the identified differentially hydroxymethylated loci drive biological effects or are biomarkers for metabolic disease, senescence, or epigenetic drift [72] remains to be established. Our hMeDIP-seq and complementary mRNA-seq data were obtained from comparable but different pigs within the corresponding groups. This integrative analysis focused on gene sets; thus, the dynamic and intricate nature of the 5hmC epigenetic mark precludes unequivocal assignment [32] of activation status to the modified loci [42]. Furthermore, obesity/dyslipidemia-driven alterations in MSCs are likely regulated by parallel epigenetic mechanisms, including micro-RNAs [15, 18, 21] and histone post-translational modifications [27], that may obscure relationships between 5hmC level and gene expression [42]; protein expression patterns of candidate genes may be further obscured by downstream post-translational regulation. Moreover, average 5hmC peak content was analyzed only in exons, not in promoters [34] or enhancers [35]. Our swine model was limited by the small sample size, short disease duration, and use of only female pigs. The model also does not permit elucidation of the relative contributions of co-morbidities to the alterations observed in MSCs. Specifically, given that our experimental pigs and patients had both obesity and dyslipidemia, the observed epigenetic alterations cannot be unequivocally attributed to one or the other [27, 84, 85]; further studies are needed to fully address this question. Harvested primary MSCs were expanded for only 3–4 passages [8, 27] to avoid the effects of long-term culture on senescence phenotype and epigenome [86]. Finally, because the growth medium used in our cell studies contains vitamin-C (2.5 μg/mL ascorbic acid phosphate), the effect of vitamin-C might have been underestimated. Future studies are needed to determine the full effect of vitamin-C on dynamic 5hmC modifications in MSCs, especially in those from human subjects with dyslipidemia, with or without obesity.

## Conclusion

We interrogated the dysregulated 5hmC profile of senescent MSCs derived from subcutaneous adipose tissue of obese pigs and identified locus-specific changes in pathways of cell proliferation and fate. Vitamin-C reverses some 5hmC changes in genes corresponding to these pathways. Importantly, we extended our proof-of-concept from swine to human MSCs. Further studies are needed to explore the diagnostic utility of the 5hmC profile as a molecular readout of MSC dysfunction in obesity and the role of vitamin-C as an epigenetic modulator of this diseased state, to improve repair potency for autologous transplantation in obese patients.

## Supplementary Information

**Additional file 1**. Supplementary tables.

**Additional file 2: Figure S1**. Tri-lineage differentiation of swine Obese and Lean-MSCs. 16 weeks of diet-induced obesity skews the tri-lineage differentiation potential of swine MSCs toward adipocytes (FABP4, red; 20X magnification) and osteocytes (osteocalcin, red; 20X magnification) and away from chondrocytes (aggrecan, red; 40X magnification).

**Additional file 3: Figure S2**. Protein expression of differentially hydroxymethylated candidate genes with functions related to apoptosis, senescence, or cell proliferation. **A** Protein levels were evaluated by Western blotting in swine Lean- and Obese-MSCs*, n* = 6 each; **B** quantification revealed decreased levels of the apoptosis-related genes CHOP/DDIT3 and CASP14 in Obese-MSCs compared with Lean-MSCs. The rightmost lane is excluded from quantification of the Western blot for CASP14, due to an obstructive artifact. Displayed Western blot images have been cropped, and group annotations are shown by white or gray horizontal bars above the images. Full-length blots/gels are presented in Figure S3. *p-value ≤ 0.05 vs. Lean-MSCs.

**Additional file 4: Figure S3**. Uncropped full-length Western blot images for swine Lean- and Obese-MSCs. Proteins levels of **A** CDKN2C and **B** CHOP/DDIT3 were normalized to **C** GAPDH loading control, while proteins levels of **D** CASP14, **E** TERT, and **F** MAPK10 were normalized to **G** GAPDH loading control. Original, unprocessed images of gels/blots are shown. All cropping margins for Figure S2 are marked with dashed blue lines, and group annotations are shown by white or gray horizontal bars above the images.

**Additional file 5: Figure S4**. Obesity associates with dysregulated 5hmC levels in swine MSCs on genes related to cell population proliferation. **A** Volcano plot showing differential 5hmC levels in Obese- versus Lean-MSCs for genes filtered by the Gene Ontology term for cell population proliferation. For a given gene, differential 5hmC levels entail *p*-value ≤ 0.05 and fold change (Obese-MSCs/Lean-MSCs) ≥ 1.4 or ≤ 0.7. Genes with high or low 5hmC levels in Obese-MSCs versus Lean-MSCs are represented with red or blue markers, respectively. High 5hmC genes with undetectable 5hmC levels in Lean-MSCs are presented as outliers and labeled as red triangles. **B** Heat maps of genes filtered by cell population proliferation showing higher or lower 5hmC levels in Obese-MSCs versus Lean-MSCs.

**Additional file 6: Figure S5**. Additional cell proliferation studies on swine Obese- and Lean-MSCs. Percentage cellular positivity for **A** PCNA or **B** Ki67, determined by immunofluorescent staining and fluorescence microscopy, did not differ in Obese- versus Lean-MSCs. PCNA: *p*-value = 0.66, Lean-MSCs: *n* = 6, Obese-MSCs: *n* = 5; Ki67: *p*-value = 0.89, *n* = 6 per group. **C** MTS assay showed decreased background-corrected absorbance at 490 nm in Obese-MSCs compared with Lean-MSCs, *n* = 6 per group; means of duplicate or triplicate measurements. *p-value = 0.0087 vs. Lean-MSCs.

**Additional file 7: Figure S6**. Apoptosis/cell death assay of swine Obese- and Lean-MSCs. Representative flow cytometry scatterplots of MSC apoptosis/cell death tested using Annexin-V and Sytox for swine **A** Lean- and **B** Obese-MSCs. The red panel represents live cells, the orange panel represents dead cells, and the yellow panel represents apoptotic cells.

**Additional file 8: Figure S7**. Lower rates of cell death in swine Obese-MSCs. **A** The percentage of apoptotic cells, determined by flow cytometry gated on Annexin-V^+^ and Sytox^–^, was not significantly different between swine Obese- and Lean-MSCs. **B** The percentage of dead cells, determined by flow cytometry gated on Annexin-V^+^ and Sytox^+^, was lower in Obese-MSCs compared with Lean-MSCs. **C** The percentage of live cells, determined by flow cytometry gated on Annexin-V^−^ and Sytox^–^, was higher in Obese-MSCs compared with Lean-MSCs. *n* = 6 per group; *p-value = 0.0022 vs. Lean-MSCs.

**Additional file 9: Figure S8**. Fluorescence microscopy TUNEL images in swine MSCs treated with a pro-apoptotic agent. Apoptosis was evaluated by terminal deoxynucleotidyl transferase dUTP nick-end-labeling assay in swine Lean- and Obese-MSCs treated either with staurosporine in DMSO at 20 nM for 24 h or with DMSO only. Representative images of DAPI-stained nuclei, TUNEL-labeled nuclei, and the merged channels are shown. Obese-MSCs showed attenuated development of apoptosis in response to staurosporine.

**Additional file 10: Figure S9**. Pairwise evaluation of TUNEL assay in swine MSCs treated with an apoptosis-inducing agent. Estimation plots for *n* = 5 pairwise comparisons of % TUNEL-positive cells in DMSO- versus staurosporine-treated swine **A** Lean- and **B** Obese-MSCs. *Paired *t*-test indicates a significant increase in TUNEL/DAPI ratio for Lean-MSCs*, p*-value = 0.0085, but not for Obese-MSCs*, p*-value = 0.44, after staurosporine treatment.

**Additional file 11: Figure S10**. Obesity-driven dysregulation of 5hmC levels on genes related to apoptotic process and cellular senescence in human MSCs. Volcano plots showing differential 5hmC levels in human Obese- versus Lean-MSCs for genes filtered by **A** the GO term for apoptotic process or **B** the union of the GO term and REACTOME Superpath for cellular senescence. For a given gene, differential 5hmC levels entail *p* ≤ 0.05 and fold change ≥ 1.4 or ≤ 0.7. Genes with high or low 5hmC levels in Obese-MSCs versus Lean-MSCs are represented with red or blue markers, respectively. Genes with very high fold change in 5hmC levels of Obese- versus Lean-MSCs are presented as outliers with triangle markers.

**Additional file 12: Figure S11**. Obesity-driven dysregulation of 5hmC levels on genes related to cell population proliferation in human MSCs. **A** Volcano plot showing differential 5hmC levels in human Obese- versus Lean-MSCs for genes filtered by the GO term for cell population proliferation. For a given gene, differential 5hmC levels entail *p*-value ≤ 0.05 and fold change ≥ 1.4 or ≤ 0.7. Genes with high or low 5hmC levels in Obese-MSCs versus Lean-MSCs are represented with red or blue markers, respectively. **B** Heat maps of genes filtered by cell population proliferation showing lower or higher 5hmC levels in human Obese-MSCs versus human Lean-MSCs.

## Data Availability

The hMeDIP-seq datasets generated and analyzed during this study can be accessed through the Gene Expression Omnibus (GEO) database with the following accession numbers: GSE216950 (for swine MSCs) and GSE216948 (for human MSCs), under the SuperSeries record GSE216953. All other data generated or analyzed during this study are included in this published article [and its supplementary information files].

## Abbreviations

5hmC: 5-Hydroxymethylcytosine
5mC: 5-Methylcytosine
BCL3: BCL3 transcription coactivator
BMI: Body mass index
CALCRL: Calcitonin receptor-like receptor
CASP: Caspase
CDK: Cyclin-dependent kinase
CDKN: Cyclin-dependent kinase inhibitor
CHOP: C/EBP homologous protein
DDIT3: DNA damage-inducible transcript 3
DIP: DNA immunoprecipitation
EIF2B5: Eukaryotic translation initiation factor 2B subunit epsilon
EVs: Extracellular vesicles
FIS1: Fission, Mitochondrial 1
FST: Follistatin
GAPDH: Glyceraldehyde-3-phosphate dehydrogenase
GEO: Gene Expression Omnibus
GSEA: Gene Set Enrichment Analysis
GO: Gene Ontology
GOBP: Gene Ontology: Biological Process
HFD: High-fat diet
hMeDIP-seq: Hydroxymethylated DNA immunoprecipitation sequencing
HOMA-IR: Homeostasis model assessment of insulin resistance
IACUC: Institutional Animal Care and Use Committee
IGV: Integrative Genomics Viewer
IL23R: Interleukin 23 receptor
IRB: Institutional Review Board
ISCT: International Society for Cellular Therapy
LDL: Low-density lipoprotein
MADD: MAP kinase activating death domain
MAP2K3: Mitogen-activated protein kinase kinase 3
MAP3K3: Mitogen-activated protein kinase kinase kinase 3
MAPK: Mitogen-activated protein kinase
MetS: Metabolic syndrome
MGI: Mouse Genome Informatics
MSC(s): Mesenchymal stem/stromal cell(s)
MSigDB: Molecular Signatures Database
MTS: (3-(4,5-Dimethylthiazol-2-yl)-5-(3-carboxymethoxyphenyl)-2-(4-sulfophenyl)-2H-tetrazolium)
NES: Normalized enrichment score
NF-κ-B: Nuclear Factor Kappa B
PCNA: Proliferating cell nuclear antigen
POT1: Protection of telomeres 1
SA-β-Gal: Senescence-associated β-galactosidase
STRING: Search Tool for the Retrieval of Interacting Genes
TERT: Telomerase reverse transcriptase
TET: Ten-eleven translocation
TNF: Tumor necrosis factor
TP53: Tumor protein P53
TUNEL: Terminal deoxynucleotidyl transferase dUTP nick-end-labeling
TWIST1: Twist Family BHLH Transcription Factor 1
