# Supplementary material for: Obesity and dyslipidemia are associated with partially reversible modifications to DNA hydroxymethylation of apoptosis- and senescence-related genes in swine adipose-derived mesenchymal stem/stromal cells
Source: Stem Cell Res Ther. 2023 May 25;14:143. doi: 10.1186/s13287-023-03372-x (PMC10214739; doi:10.1186/s13287-023-03372-x)
Supplement: Supplementary file 1 — Additional file 1. Supplementary tables. [file 13287_2023_3372_MOESM1_ESM.pdf]

SUPPLEMENTAL MATERIAL

"Obesity and dyslipidemia are associated with partially reversible modifications to DNA hydroxymethylation of apoptosis- and senescence-related genes in swine adipose-derived mesenchymal stem/stromal cells"

Logan M. Glasstetter[1], Tomiwa S. Oderinde[1], Mohit Mirchandani[1], Kamalnath Sankaran Rajagopalan[1], Samer H. Barsom[1], Roman Thaler[2], Sarosh Siddiqi[1], Xiang-Yang Zhu[1], Hui Tang[1], Kyra L. Jordan[1], Ishran M. Saadiq[1], Andre J. van Wijnen[3], Alfonso Eirin[1], Lilach O. Lerman[1]

[1]Division of Nephrology and Hypertension, Mayo Clinic, Rochester, MN, USA.

[2]Department of Orthopedic Surgery, Mayo Clinic, Rochester, MN, USA.

[3]Department of Biochemistry, University of Vermont, Burlington, VT, USA.

Corresponding Author:

Lilach O. Lerman, MD, PhD, Division of Nephrology and Hypertension, Mayo Clinic, 200 First Street SW, Rochester, MN, 55905. (lerman.lilach@mayo.edu)

Phone: (507)-266-9376, Fax: (507)-266-9316

Supplemental Tables

Table S1. DAVID Functional Annotation Chart (GOTERM_BP_DIRECT) computed for genes with a multiplicity* of at least 2 in the 95 gene sets with high 5hmC/high mRNA levels identified in an integrative analysis of swine Obese- versus Lean-MSCs.†

Term	Count	%	P-Value	Bonferroni	Benjamini	FDR
GO:0060337~type I interferon signaling pathway	66	2.49	4.26E-53	3.33E-49	3.33E-49	3.06E-49
GO:0019221~cytokine-mediated signaling pathway	145	5.46	1.93E-45	1.51E-41	7.54E-42	6.94E-42
GO:0045944~positive regulation of transcription from RNA polymerase II promoter	311	11.72	3.09E-36	2.41E-32	8.05E-33	7.41E-33
GO:0006954~inflammatory response	150	5.65	2.01E-35	1.57E-31	3.93E-32	3.62E-32
GO:0006915~apoptotic process	188	7.08	1.89E-34	1.48E-30	2.96E-31	2.73E-31
GO:0001666~response to hypoxia	88	3.32	1.17E-30	9.12E-27	1.38E-27	1.27E-27
GO:0043066~negative regulation of apoptotic process	170	6.41	1.23E-30	9.64E-27	1.38E-27	1.27E-27
GO:0010628~positive regulation of gene expression	163	6.14	7.33E-29	5.73E-25	7.16E-26	6.60E-26
GO:0043065~positive regulation of apoptotic process	131	4.94	1.61E-27	1.26E-23	1.40E-24	1.29E-24
GO:0043123~positive regulation of I-kappaB kinase/NF-kappaB signaling	85	3.20	1.73E-26	1.35E-22	1.35E-23	1.25E-23
GO:0006687~glycosphingolipid metabolic process	36	1.36	2.15E-26	1.68E-22	1.53E-23	1.41E-23
GO:0007165~signal transduction	285	10.74	4.74E-26	3.70E-22	3.09E-23	2.84E-23
GO:0043312~neutrophil degranulation	149	5.61	2.32E-25	1.81E-21	1.40E-22	1.29E-22
GO:0008285~negative regulation of cell proliferation	142	5.35	1.93E-24	1.51E-20	1.08E-21	9.94E-22
GO:0071260~cellular response to mechanical stimulus	51	1.92	4.09E-24	3.20E-20	2.13E-21	1.96E-21
GO:0042493~response to drug	104	3.92	8.02E-24	6.27E-20	3.92E-21	3.61E-21
GO:0000187~activation of MAPK activity	60	2.26	2.92E-22	2.28E-18	1.34E-19	1.24E-19

GO:0045893~positive regulation of transcription, DNA-templated	178	6.71	3.77E-22	2.94E-18	1.64E-19	1.51E-19
GO:0001934~positive regulation of protein phosphorylation	82	3.09	5.92E-22	4.63E-18	2.44E-19	2.24E-19
GO:0071222~cellular response to lipopolysaccharide	78	2.94	9.83E-22	7.68E-18	3.84E-19	3.54E-19
GO:0001525~angiogenesis	91	3.43	2.18E-21	1.70E-17	8.10E-19	7.46E-19
GO:0008284~positive regulation of cell proliferation	151	5.69	3.29E-21	2.57E-17	1.17E-18	1.08E-18
GO:0051607~defense response to virus	84	3.17	1.18E-20	9.24E-17	4.02E-18	3.70E-18
GO:0071456~cellular response to hypoxia	61	2.30	1.28E-20	1.00E-16	4.18E-18	3.85E-18
GO:0030335~positive regulation of cell migration	92	3.47	1.66E-20	1.30E-16	5.19E-18	4.78E-18

*This multiplicity requirement is invoked to adjust the size of a composite list of genes (from 95 gene-sets) to a number that is viable for the overlap analysis, while prioritizing the most important genes.

†Salient Gene Ontology (GO) terms with functions related to apoptotic process, cell proliferation, defense response, or cytokine-mediated signaling are highlighted.

Table S2. DAVID Functional Annotation Chart (GOTERM_BP_DIRECT) computed for genes with a multiplicity of at least 3 in the 95 gene sets with high 5hmC/high mRNA levels identified in an integrative analysis of swine Obese- versus Lean-MSCs.

Term	Count	%	P-Value	Bonferroni	Benjamini	FDR
GO:0060337~type I interferon signaling pathway	56	5.31	1.62E-57	9.06E-54	9.06E-54	8.24E-54
GO:0019221~cytokine-mediated signaling pathway	99	9.39	1.25E-47	7.01E-44	3.50E-44	3.19E-44
GO:0006954~inflammatory response	92	8.73	5.37E-33	3.00E-29	1.00E-29	9.11E-30
GO:0006915~apoptotic process	108	10.25	2.84E-30	1.59E-26	3.97E-27	3.62E-27
GO:0061621~canonical glycolysis	24	2.28	9.06E-29	5.07E-25	1.01E-25	9.22E-26
GO:0043066~negative regulation of apoptotic process	100	9.49	1.54E-28	8.63E-25	1.44E-25	1.31E-25
GO:0001666~response to hypoxia	57	5.41	6.15E-28	3.44E-24	4.91E-25	4.47E-25
GO:0045944~positive regulation of transcription from RNA polymerase II promoter	157	14.90	1.82E-26	1.02E-22	1.27E-23	1.16E-23
GO:0009615~response to virus	43	4.08	9.46E-25	5.29E-21	5.88E-22	5.35E-22
GO:0071222~cellular response to lipopolysaccharide	54	5.12	3.02E-24	1.69E-20	1.69E-21	1.53E-21
GO:0042493~response to drug	66	6.26	5.21E-24	2.91E-20	2.65E-21	2.41E-21
GO:0043065~positive regulation of apoptotic process	76	7.21	1.20E-23	6.74E-20	5.61E-21	5.11E-21
GO:0051607~defense response to virus	57	5.41	3.40E-23	1.90E-19	1.46E-20	1.33E-20
GO:0010628~positive regulation of gene expression	89	8.44	8.38E-23	4.69E-19	3.35E-20	3.05E-20
GO:0030335~positive regulation of cell migration	60	5.69	4.76E-22	2.66E-18	1.77E-19	1.61E-19
GO:0008285~negative regulation of cell proliferation	81	7.69	1.51E-21	8.46E-18	5.29E-19	4.81E-19
GO:0006096~glycolytic process	25	2.37	2.34E-21	1.31E-17	7.69E-19	7.00E-19
GO:0071260~cellular response to mechanical stimulus	34	3.23	5.93E-21	3.31E-17	1.84E-18	1.68E-18
GO:0006687~glycosphingolipid metabolic process	24	2.28	1.42E-19	7.95E-16	4.19E-17	3.81E-17
GO:0043312~neutrophil degranulation	80	7.59	1.65E-19	9.25E-16	4.62E-17	4.21E-17

GO:0008284~positive regulation of cell proliferation	84	7.97	7.97E-19	4.46E-15	2.12E-16	1.93E-16
GO:0007165~signal transduction	137	13.00	1.32E-17	7.35E-14	3.34E-15	3.04E-15
GO:0010832~negative regulation of myotube differentiation	15	1.42	1.92E-17	1.07E-13	4.66E-15	4.24E-15
GO:0045893~positive regulation of transcription, DNA-templated	92	8.73	5.80E-17	6.21E-13	1.35E-14	1.23E-14
GO:0071456~cellular response to hypoxia	37	3.51	8.49E-17	6.21E-13	1.86E-14	1.69E-14

Table S3. DAVID Functional Annotation Chart (GOTERM_BP_DIRECT) computed for genes with a multiplicity of at least 4 in the 95 gene sets with high 5hmC/high mRNA levels identified in an integrative analysis of swine Obese- versus Lean-MSCs.

Term	Count	%	P-Value	Bonferroni	Benjamini	FDR
GO:0060337~type I interferon signaling pathway	32	6.81	4.21E-32	1.66E-28	1.66E-28	1.50E-28
GO:0019221~cytokine-mediated signaling pathway	53	11.28	5.14E-28	2.03E-24	1.01E-24	9.17E-25
GO:0061621~canonical glycolysis	20	4.26	4.71E-27	1.86E-23	6.19E-24	5.60E-24
GO:0006954~inflammatory response	54	11.49	3.19E-24	1.26E-20	3.15E-21	2.85E-21
GO:0045944~positive regulation of transcription from RNA polymerase II promoter	92	19.57	3.05E-23	1.20E-19	2.38E-20	2.16E-20
GO:0006915~apoptotic process	63	13.40	3.63E-23	1.43E-19	2.38E-20	2.16E-20
GO:0010832~negative regulation of myotube differentiation	15	3.19	2.67E-22	1.05E-18	1.50E-19	1.36E-19
GO:0043066~negative regulation of apoptotic process	59	12.55	3.05E-22	1.20E-18	1.50E-19	1.36E-19
GO:0001666~response to hypoxia	36	7.66	8.47E-22	3.34E-18	3.71E-19	3.36E-19
GO:0009615~response to virus	29	6.17	1.04E-20	4.09E-17	4.09E-18	3.70E-18
GO:0008285~negative regulation of cell proliferation	52	11.06	3.93E-20	1.55E-16	1.41E-17	1.28E-17
GO:0006096~glycolytic process	19	4.04	2.05E-19	8.07E-16	6.72E-17	6.09E-17
GO:0043065~positive regulation of apoptotic process	46	9.79	5.67E-19	2.24E-15	1.72E-16	1.56E-16
GO:0071222~cellular response to lipopolysaccharide	33	7.02	3.06E-18	1.21E-14	8.61E-16	7.80E-16
GO:0010628~positive regulation of gene expression	52	11.06	7.30E-18	2.88E-14	1.92E-15	1.74E-15
GO:0030335~positive regulation of cell migration	36	7.66	8.90E-17	4.38E-13	2.19E-14	1.99E-14
GO:0045893~positive regulation of transcription, DNA-templated	57	12.13	3.56E-16	1.31E-12	8.27E-14	7.48E-14
GO:0071260~cellular response to mechanical stimulus	22	4.68	3.84E-16	1.31E-12	8.40E-14	7.61E-14
GO:0048661~positive regulation of smooth muscle cell proliferation	20	4.26	1.44E-15	5.69E-12	2.98E-13	2.70E-13

GO:0043312~neutrophil degranulation	47	10.00	1.56E-15	6.13E-12	3.07E-13	2.78E-13
GO:0042493~response to drug	36	7.66	2.66E-15	1.05E-11	4.79E-13	4.34E-13
GO:0051607~defense response to virus	32	6.81	2.68E-15	1.05E-11	4.79E-13	4.34E-13
GO:0060333~interferon-gamma-mediated signaling pathway	20	4.26	4.57E-15	1.79E-11	7.52E-13	6.81E-13
GO:0008284~positive regulation of cell proliferation	49	10.43	4.58E-15	1.79E-11	7.52E-13	6.81E-13
GO:0007165~signal transduction	77	16.38	8.08E-15	3.19E-11	1.27E-12	1.15E-12

Table S4. DAVID Functional Annotation Chart (GOTERM_BP_DIRECT) computed for genes with a multiplicity of at least 5 in the 95 gene sets with high 5hmC/high mRNA levels identified in an integrative analysis of swine Obese- versus Lean-MSCs.

Term	Count	%	P-Value	Bonferroni	Benjamini	FDR
GO:0061621~canonical glycolysis	17	7.26	7.81E-26	2.30E-22	2.30E-22	2.06E-22
GO:0006954~inflammatory response	41	17.52	3.42E-25	1.01E-21	5.05E-22	4.51E-22
GO:0045944~positive regulation of transcription from RNA polymerase II promoter	63	26.92	2.00E-23	5.92E-20	1.97E-20	1.76E-20
GO:0043066~negative regulation of apoptotic process	40	17.09	8.30E-20	2.45E-16	6.12E-17	5.47E-17
GO:0019221~cytokine-mediated signaling pathway	32	13.68	3.06E-19	9.04E-16	1.81E-16	1.62E-16
GO:0071222~cellular response to lipopolysaccharide	26	11.11	5.33E-19	1.57E-15	2.62E-16	2.34E-16
GO:0045893~positive regulation of transcription, DNA-templated	43	18.38	7.06E-19	2.08E-15	2.98E-16	2.66E-16
GO:0008285~negative regulation of cell proliferation	36	15.38	1.83E-18	5.40E-15	6.43E-16	5.75E-16
GO:0006915~apoptotic process	40	17.09	1.96E-18	5.78E-15	6.43E-16	5.75E-16
GO:0001666~response to hypoxia	25	10.68	2.64E-18	7.78E-15	7.78E-16	6.96E-16
GO:0060337~type I interferon signaling pathway	18	7.69	4.33E-18	1.28E-14	1.16E-15	1.04E-15
GO:0006096~glycolytic process	15	6.41	1.03E-17	3.05E-14	2.54E-15	2.27E-15
GO:0042493~response to drug	26	11.11	1.02E-14	3.02E-11	2.32E-12	2.07E-12
GO:0010628~positive regulation of gene expression	33	14.10	2.69E-14	7.93E-11	5.67E-12	5.07E-12
GO:1902895~positive regulation of pri-miRNA transcription from RNA polymerase II promoter	13	5.56	1.90E-13	5.60E-10	3.72E-11	3.33E-11
GO:0035994~response to muscle stretch	10	4.27	2.02E-13	5.96E-10	3.72E-11	3.33E-11
GO:0008284~positive regulation of cell proliferation	32	13.68	4.74E-13	1.40E-09	8.24E-11	7.36E-11
GO:0043312~neutrophil degranulation	30	12.82	1.03E-12	3.04E-09	1.63E-10	1.45E-10
GO:0000187~activation of MAPK activity	17	7.26	1.05E-12	3.09E-09	1.63E-10	1.45E-10

GO:0010832~negative regulation of myotube differentiation	9	3.85	2.35E-12	6.93E-09	3.46E-10	3.10E-10
GO:0030335~positive regulation of cell migration	22	9.40	6.05E-12	1.79E-08	8.51E-10	7.61E-10
GO:0009615~response to virus	16	6.84	7.50E-12	2.21E-08	1.01E-09	8.99E-10
GO:1903800~positive regulation of production of miRNAs involved in gene silencing by miRNA	8	3.42	2.52E-11	7.45E-08	3.24E-09	2.90E-09
GO:0051607~defense response to virus	20	8.55	2.67E-11	7.89E-08	3.29E-09	2.94E-09
GO:0001934~positive regulation of protein phosphorylation	19	8.12	5.93E-11	1.75E-07	7.01E-09	6.26E-09

Table S5. DAVID Functional Annotation Chart (GOTERM_BP_DIRECT) computed for genes with a multiplicity of at least 6 in the 95 gene sets with high 5hmC/high mRNA levels identified in an integrative analysis of swine Obese- versus Lean-MSCs.

Term	Count	%	P-Value	Bonferroni	Benjamini	FDR
GO:0019221~cytokine-mediated signaling pathway	26	19.85	1.08E-19	2.36E-16	9.66E-17	8.69E-17
GO:0043066~negative regulation of apoptotic process	31	23.66	1.32E-19	2.89E-16	9.66E-17	8.69E-17
GO:0006954~inflammatory response	28	21.37	1.32E-19	2.90E-16	9.66E-17	8.69E-17
GO:0071222~cellular response to lipopolysaccharide	21	16.03	1.57E-18	3.45E-15	8.63E-16	7.76E-16
GO:0045944~positive regulation of transcription from RNA polymerase II promoter	40	30.53	3.49E-17	7.66E-14	1.53E-14	1.38E-14
GO:0008285~negative regulation of cell proliferation	26	19.85	3.98E-16	9.74E-13	1.46E-13	1.31E-13
GO:0001666~response to hypoxia	17	12.98	9.22E-14	2.02E-10	2.89E-11	2.60E-11
GO:0035994~response to muscle stretch	9	6.87	1.38E-13	3.03E-10	3.79E-11	3.41E-11
GO:0030335~positive regulation of cell migration	18	13.74	1.67E-12	3.67E-09	4.07E-10	3.66E-10
GO:0061621~canonical glycolysis	9	6.87	2.24E-12	4.92E-09	4.48E-10	4.03E-10
GO:0045893~positive regulation of transcription, DNA-templated	26	19.85	2.25E-12	4.93E-09	4.48E-10	4.03E-10
GO:0060337~type I interferon signaling pathway	11	8.40	3.21E-11	7.04E-08	5.87E-09	5.28E-09
GO:0006915~apoptotic process	23	17.56	5.03E-11	1.10E-07	8.48E-09	7.63E-09
GO:0042493~response to drug	17	12.98	9.16E-11	2.01E-07	1.44E-08	1.29E-08
GO:0000187~activation of MAPK activity	12	9.16	4.68E-10	1.03E-06	6.84E-08	6.15E-08
GO:0008284~positive regulation of cell proliferation	21	16.03	5.09E-10	1.12E-06	6.98E-08	6.28E-08
GO:1902895~positive regulation of pri-miRNA transcription from RNA polymerase II promoter	9	6.87	7.04E-10	1.54E-06	9.07E-08	8.15E-08
GO:0048661~positive regulation of smooth muscle cell proliferation	10	7.63	7.44E-10	1.63E-06	9.07E-08	8.15E-08
GO:0010628~positive regulation of gene expression	20	15.27	1.74E-09	3.82E-06	2.01E-07	1.81E-07

GO:0071356~cellular response to tumor necrosis factor	12	9.16	2.88E-09	6.32E-06	3.16E-07	2.84E-07
GO:0051607~defense response to virus	14	10.69	3.37E-09	7.39E-06	3.52E-07	3.17E-07
GO:0071260~cellular response to mechanical stimulus	10	7.63	4.56E-09	1.00E-05	4.36E-07	3.92E-07
GO:0031663~lipopolysaccharide-mediated signaling pathway	8	6.11	4.57E-09	1.00E-05	4.36E-07	3.92E-07
GO:0009615~response to virus	11	8.40	4.98E-09	1.09E-05	4.55E-07	4.09E-07
GO:0006096~glycolytic process	8	6.11	6.75E-09	1.48E-05	5.93E-07	5.33E-07

Table S6. DAVID Functional Annotation Chart (GOTERM_BP_DIRECT) computed for genes from 7 gene sets with low 5hmC/low mRNA levels identified in an integrative analysis of swine Obese- versus Lean-MSCs.‡

Term	Count	%	P-Value	Bonferroni	Benjamini	FDR
GO:0045494~photoreceptor cell maintenance	44	8.19	2.61E-66	8.09E-63	8.09E-63	7.92E-63
GO:0001916~positive regulation of T cell mediated cytotoxicity	25	4.66	2.64E-36	8.18E-33	4.09E-33	4.00E-33
GO:0045954~positive regulation of natural killer cell mediated cytotoxicity	24	4.47	8.12E-34	2.52E-30	8.39E-31	8.20E-31
GO:0032729~positive regulation of interferon-gamma production	18	3.35	2.13E-11	6.60E-08	1.65E-08	1.61E-08
GO:0050776~regulation of immune response	25	4.66	3.15E-09	9.75E-06	1.95E-06	1.91E-06
GO:0002486~antigen processing and presentation of endogenous peptide antigen via MHC class I via ER pathway, TAP-independent	7	1.30	9.03E-09	2.80E-05	4.67E-06	4.56E-06
GO:0007268~chemical synaptic transmission	27	5.03	1.47E-08	4.56E-05	6.51E-06	6.37E-06
GO:0002480~antigen processing and presentation of exogenous peptide antigen via MHC class I, TAP-independent	7	1.30	2.65E-08	8.21E-05	9.12E-06	8.92E-06
GO:0050953~sensory perception of light stimulus	7	1.30	2.65E-08	8.21E-05	9.12E-06	8.92E-06
GO:0007601~visual perception	23	4.28	3.21E-08	9.96E-05	9.96E-06	9.74E-06

‡Salient Gene Ontology (GO) terms with functions related to leukocyte-mediated cytotoxicity are highlighted.

Table S7. Laboratory measures and demographic data of Lean and Obese patients ($n = 5$, each).

	**Lean**	**Obese**
**Demographics**:		
Age (years)	60.4 ± 7.2	56.6 ± 9.5
Sex (Male/Female)	2/3	2/3
Body Mass Index (kg/m²)	24.8 (24.3-26.8)	45.9 (39.4-48.3)*
**Relevant laboratory measures**:		
Mean blood pressure (mmHg)	82.4 ± 12.6	91.5 ± 7.9
Total cholesterol (mg/dL)	198.3 ± 25.4	209.5 ± 7.8
Triglycerides (mg/dL)	73.6 ± 8.3	111.5 ± 10.6*
LDL (mg/dL)	128.3 ± 8.7	125.5 ± 4.9
Fasting glucose (mg/dL)	92.0 (83.0-118.5)	96.0 (89.0-137.5)

*LDL: low-density lipoprotein. Data displayed as mean ± standard deviation, or median (interquartile range). * $p \leq 0.05$ vs. Lean.*
