# Supplementary material for: Obesity and dyslipidemia are associated with partially reversible modifications to DNA hydroxymethylation of apoptosis- and senescence-related genes in swine adipose-derived mesenchymal stem/stromal cells
Source: Stem Cell Res Ther. 2023 May 25;14:143. doi: 10.1186/s13287-023-03372-x (PMC10214739; doi:10.1186/s13287-023-03372-x)
Supplement: Supplementary file 2 — Additional file 2: Figure S1. Tri-lineage differentiation of swine Obese and Lean-MSCs. 16 weeks of diet-induced obesity skews the tri-lineage differentiation potential of swine MSCs toward adipocytes (FABP4, red; 20X magnification) and osteocytes (osteocalcin, red; 20X magnification) and away from chondrocytes (aggrecan, red; 40X magnification). [file 13287_2023_3372_MOESM2_ESM.pdf]

Fig. S1

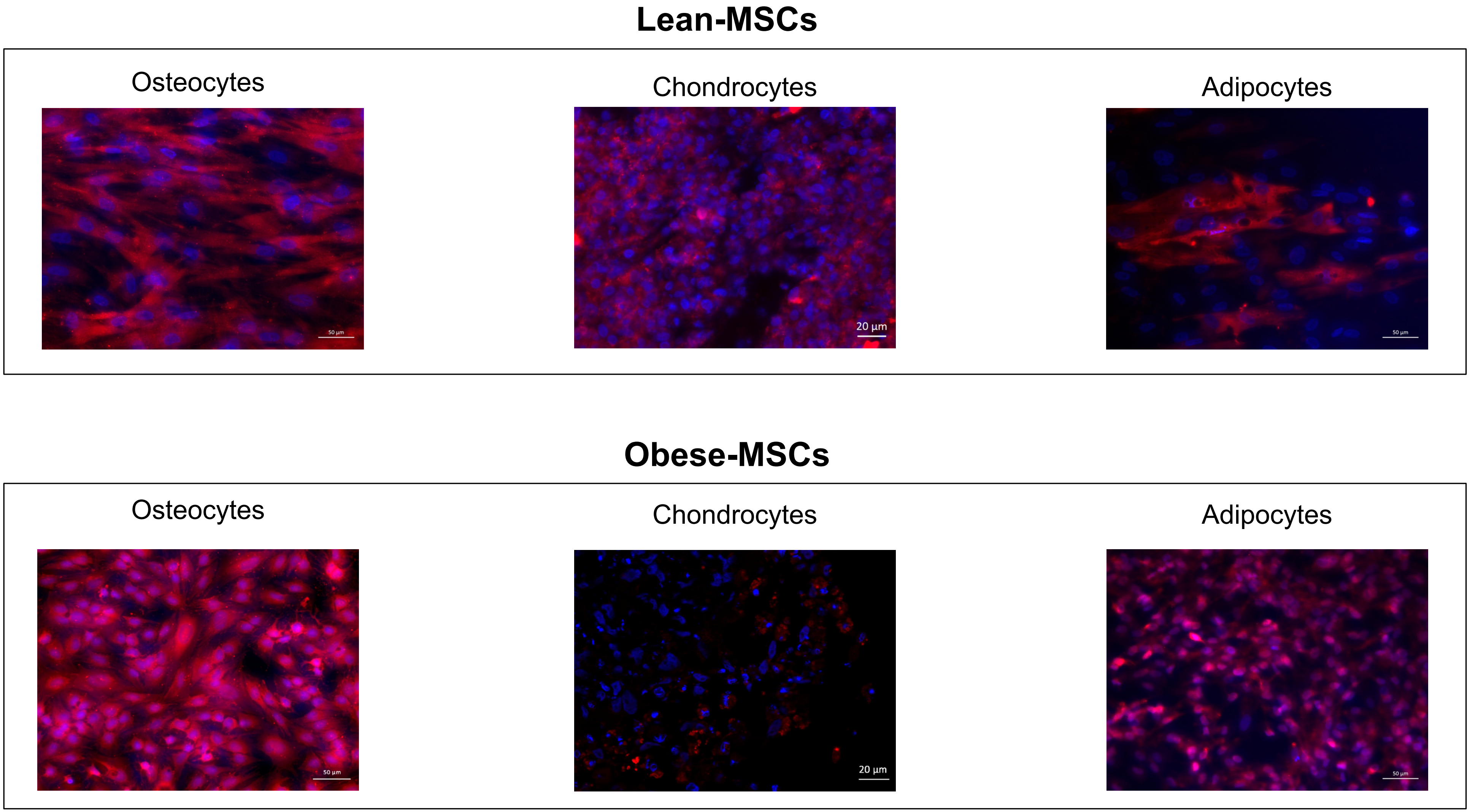
Lean-MSCs
Osteocytes
Chondrocytes
Adipocytes
50 µm
20 µm
50 µm
Obese-MSCs
Osteocytes
Chondrocytes
Adipocytes
50 µm
20 µm
50 µm
