# Supplementary figures and images for: Obesity and dyslipidemia are associated with partially reversible modifications to DNA hydroxymethylation of apoptosis- and senescence-related genes in swine adipose-derived mesenchymal stem/stromal cells
Source: Stem Cell Res Ther. 2023 May 25;14:143. doi: 10.1186/s13287-023-03372-x (PMC10214739; doi:10.1186/s13287-023-03372-x)

Fig. S2

**A**

Lean-MSCs  
Obese-MSCs

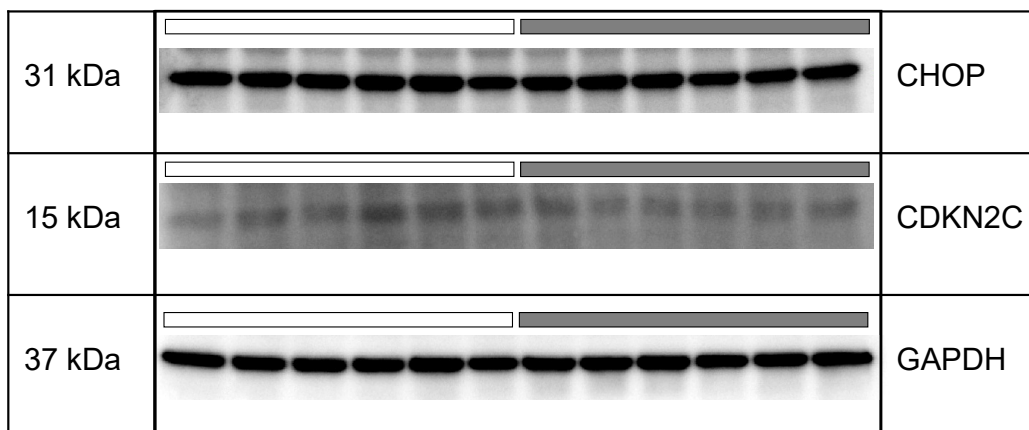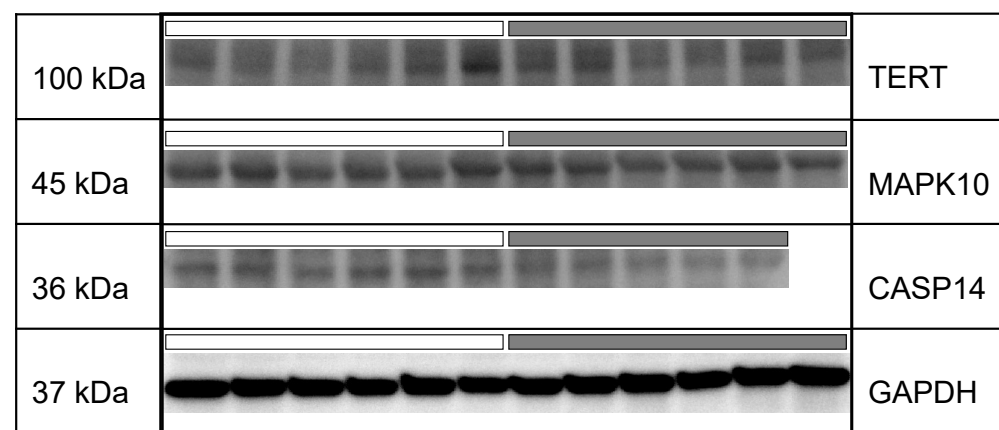**B**

CHOP/GAPDH

CDKN2C/GAPDH

TERT/GAPDH

CASP14/GAPDH

MAPK10/GAPDH

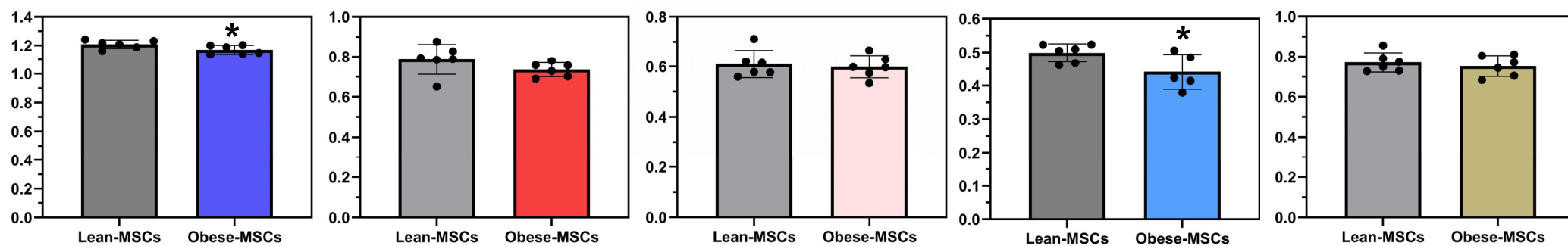

Supplement: Supplementary file 3 — Additional file 3: Figure S2. Protein expression of differentially hydroxymethylated candidate genes with functions related to apoptosis, senescence, or cell proliferation. A Protein levels were evaluated by Western blotting in swine Lean- and Obese-MSCs, n = 6 each; B quantification revealed decreased levels of the apoptosis-related genes CHOP/DDIT3 and CASP14 in Obese-MSCs compared with Lean-MSCs. The rightmost lane is excluded from quantification of the Western blot for CASP14, due to an obstructive artifact. Displayed Western blot images have been cropped, and group annotations are shown by white or gray horizontal bars above the images. Full-length blots/gels are presented in Figure S3. *p-value ≤ 0.05 vs. Lean-MSCs. [file 13287_2023_3372_MOESM3_ESM.pdf]

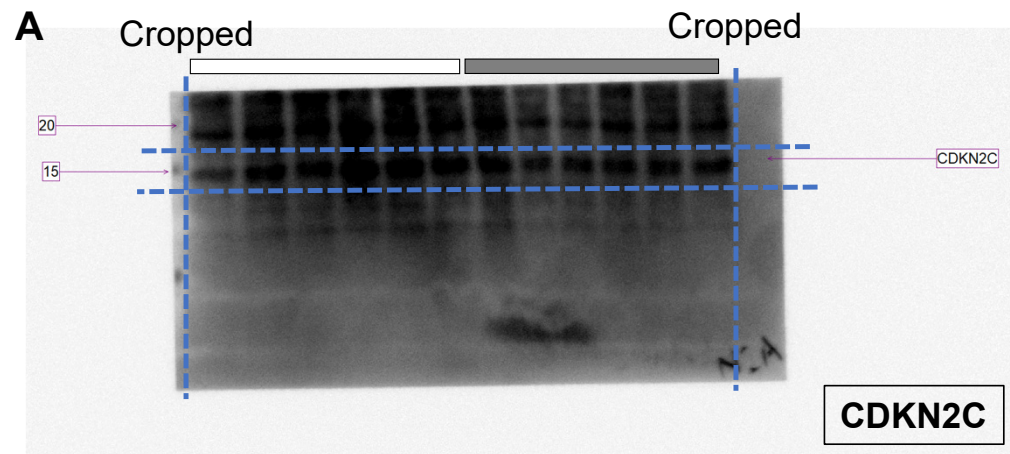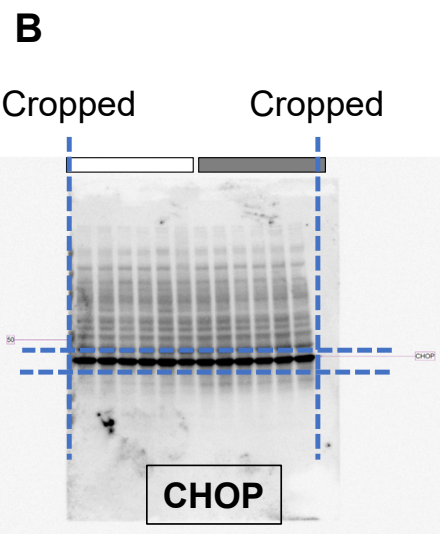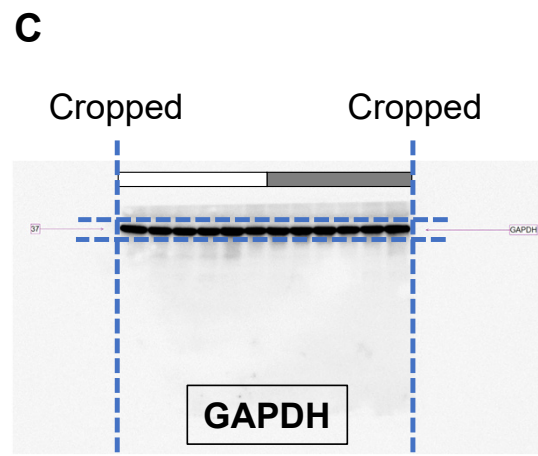

Lean-MSCs

Obese-MSCs

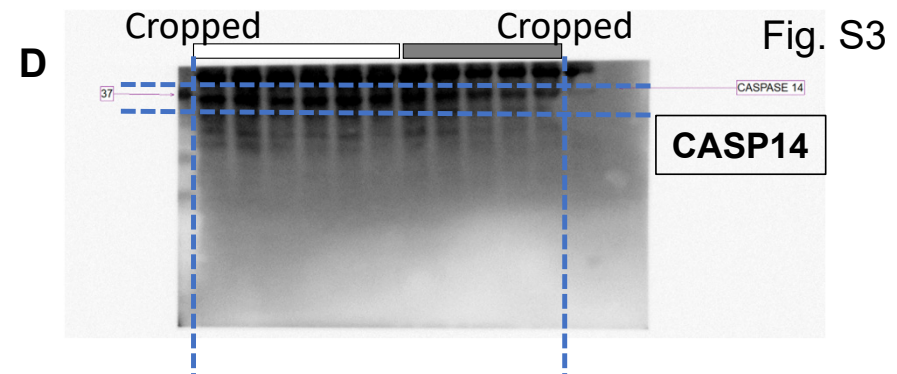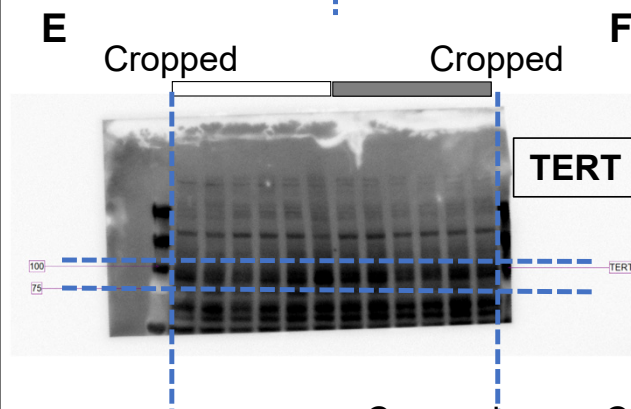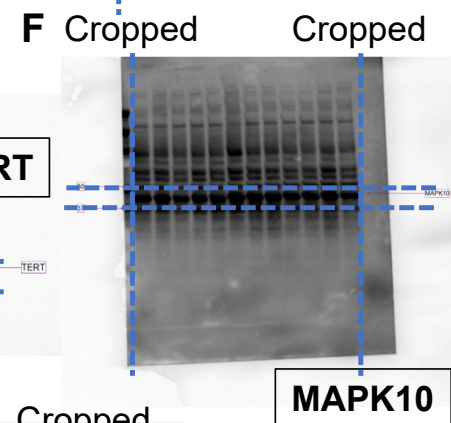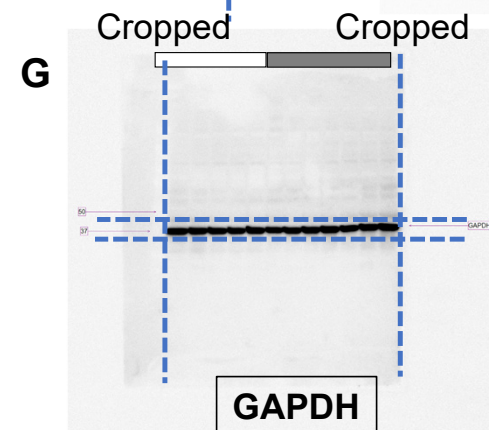

Fig. S3

Supplement: Supplementary file 4 — Additional file 4: Figure S3. Uncropped full-length Western blot images for swine Lean- and Obese-MSCs. Proteins levels of A CDKN2C and B CHOP/DDIT3 were normalized to C GAPDH loading control, while proteins levels of D CASP14, E TERT, and F MAPK10 were normalized to G GAPDH loading control. Original, unprocessed images of gels/blots are shown. All cropping margins for Figure S2 are marked with dashed blue lines, and group annotations are shown by white or gray horizontal bars above the images. [file 13287_2023_3372_MOESM4_ESM.pdf]

# Swine MSCs

**A**

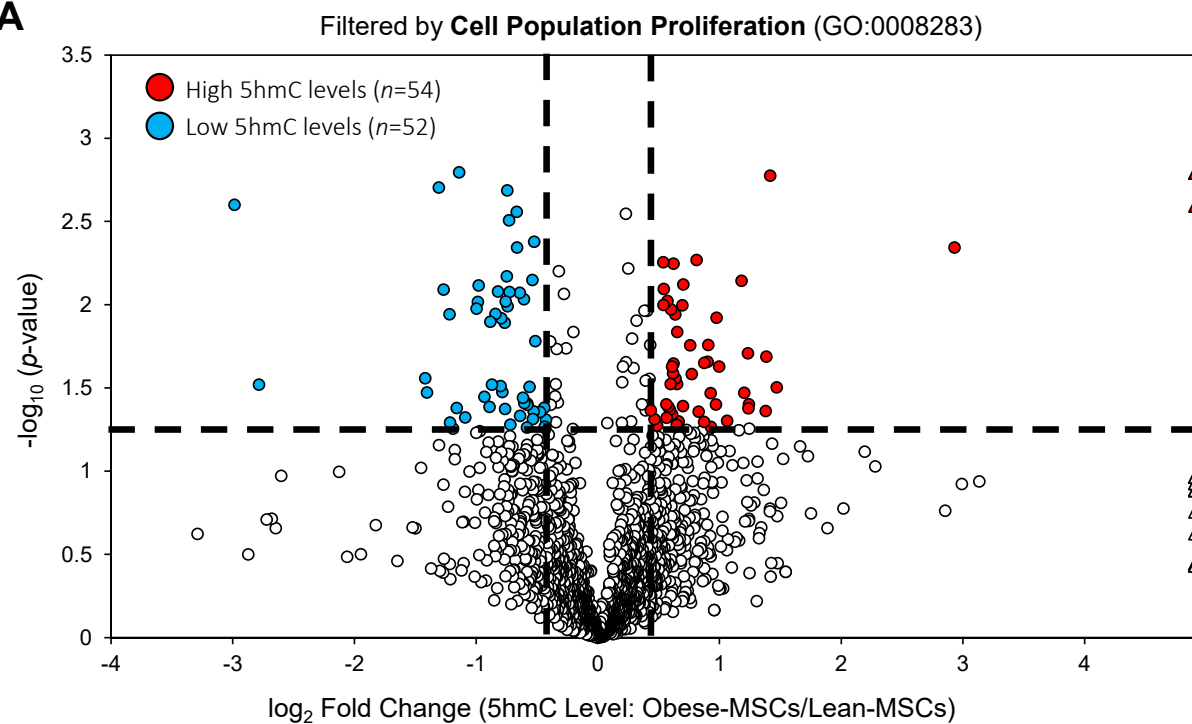

Lean-MSCs  
Obese-MSCs

**B**

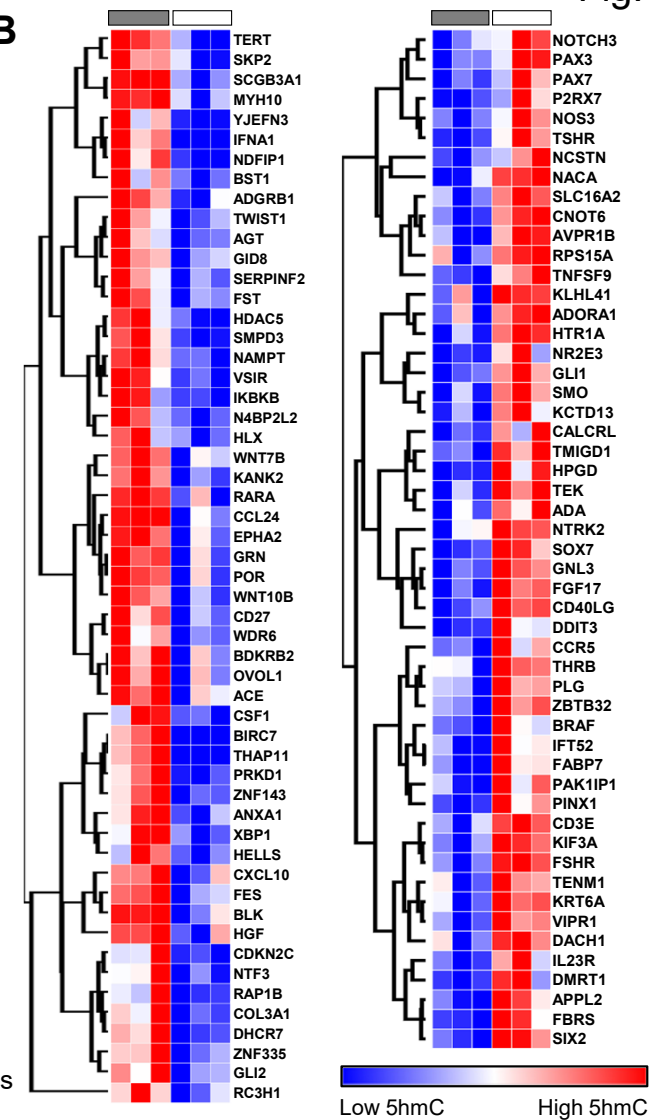

Fig. S4

Supplement: Supplementary file 5 — Additional file 5: Figure S4. Obesity associates with dysregulated 5hmC levels in swine MSCs on genes related to cell population proliferation. A Volcano plot showing differential 5hmC levels in Obese- versus Lean-MSCs for genes filtered by the Gene Ontology term for cell population proliferation. For a given gene, differential 5hmC levels entail p-value ≤ 0.05 and fold change (Obese-MSCs/Lean-MSCs) ≥ 1.4 or ≤ 0.7. Genes with high or low 5hmC levels in Obese-MSCs versus Lean-MSCs are represented with red or blue markers, respectively. High 5hmC genes with undetectable 5hmC levels in Lean-MSCs are presented as outliers and labeled as red triangles. B Heat maps of genes filtered by cell population proliferation showing higher or lower 5hmC levels in Obese-MSCs versus Lean-MSCs. [file 13287_2023_3372_MOESM5_ESM.pdf]

Fig. S7

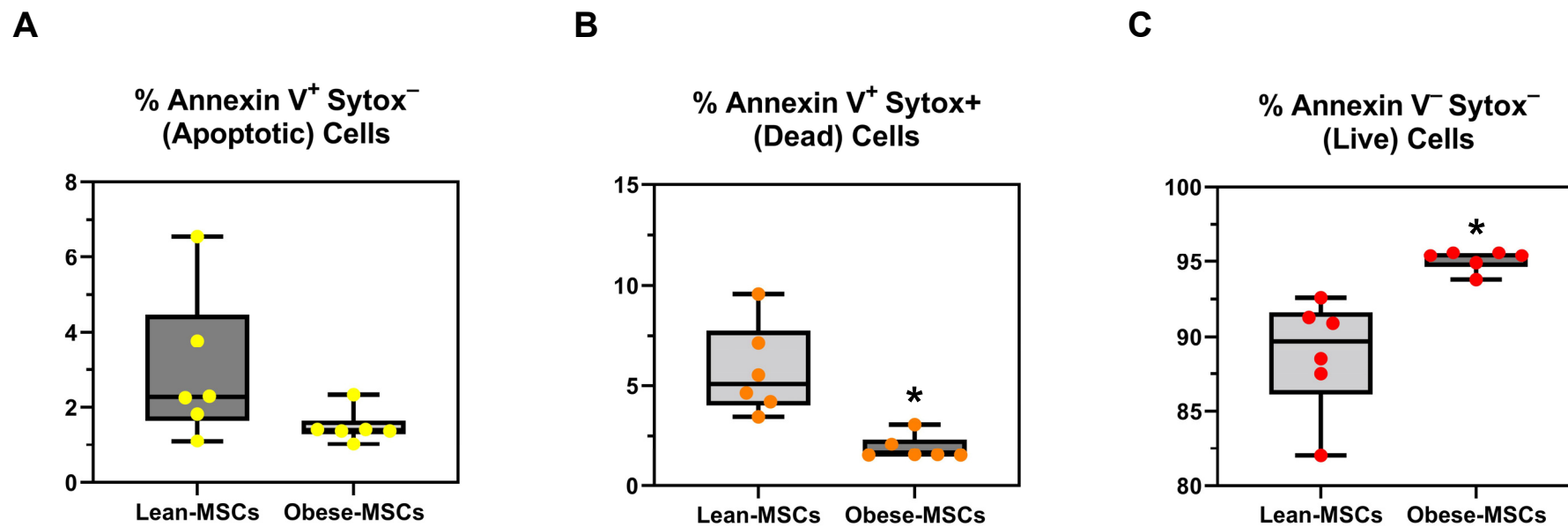

Supplement: Supplementary file 8 — Additional file 8: Figure S7. Lower rates of cell death in swine Obese-MSCs. A The percentage of apoptotic cells, determined by flow cytometry gated on Annexin-V+ and Sytox–, was not significantly different between swine Obese- and Lean-MSCs. B The percentage of dead cells, determined by flow cytometry gated on Annexin-V+ and Sytox+, was lower in Obese-MSCs compared with Lean-MSCs. C The percentage of live cells, determined by flow cytometry gated on Annexin-V− and Sytox–, was higher in Obese-MSCs compared with Lean-MSCs. n = 6 per group; *p-value = 0.0022 vs. Lean-MSCs. [file 13287_2023_3372_MOESM8_ESM.pdf]

Fig. S9

**A**

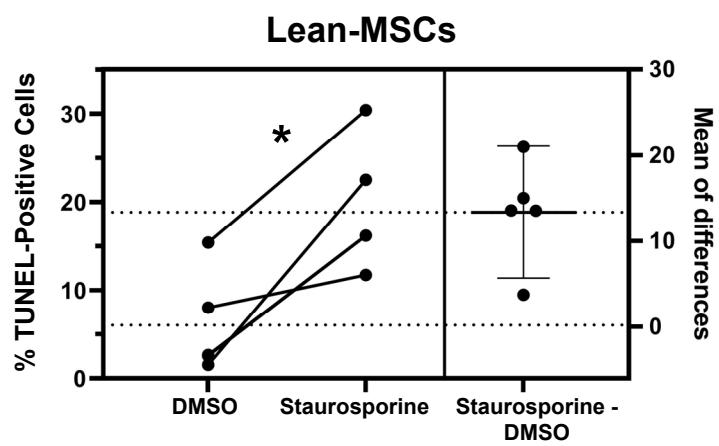

**B**

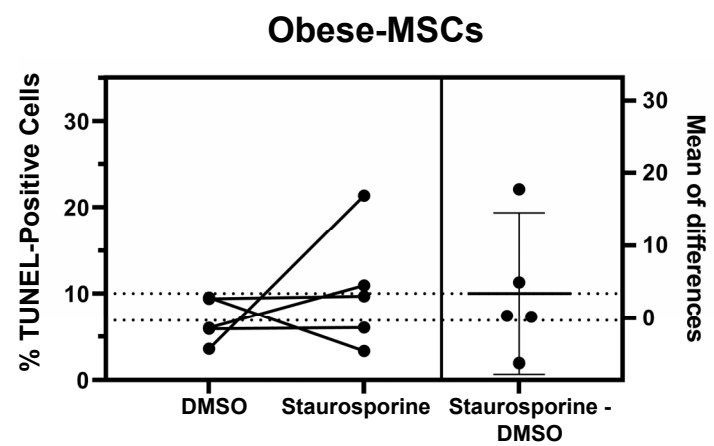

Supplement: Supplementary file 10 — Additional file 10: Figure S9. Pairwise evaluation of TUNEL assay in swine MSCs treated with an apoptosis-inducing agent. Estimation plots for n = 5 pairwise comparisons of % TUNEL-positive cells in DMSO- versus staurosporine-treated swine A Lean- and B Obese-MSCs. *Paired t-test indicates a significant increase in TUNEL/DAPI ratio for Lean-MSCs, p-value = 0.0085, but not for Obese-MSCs, p-value = 0.44, after staurosporine treatment. [file 13287_2023_3372_MOESM10_ESM.pdf]

Fig. S11

## Human MSCs

A

Filtered by Cell Population Proliferation (GO:0008283)

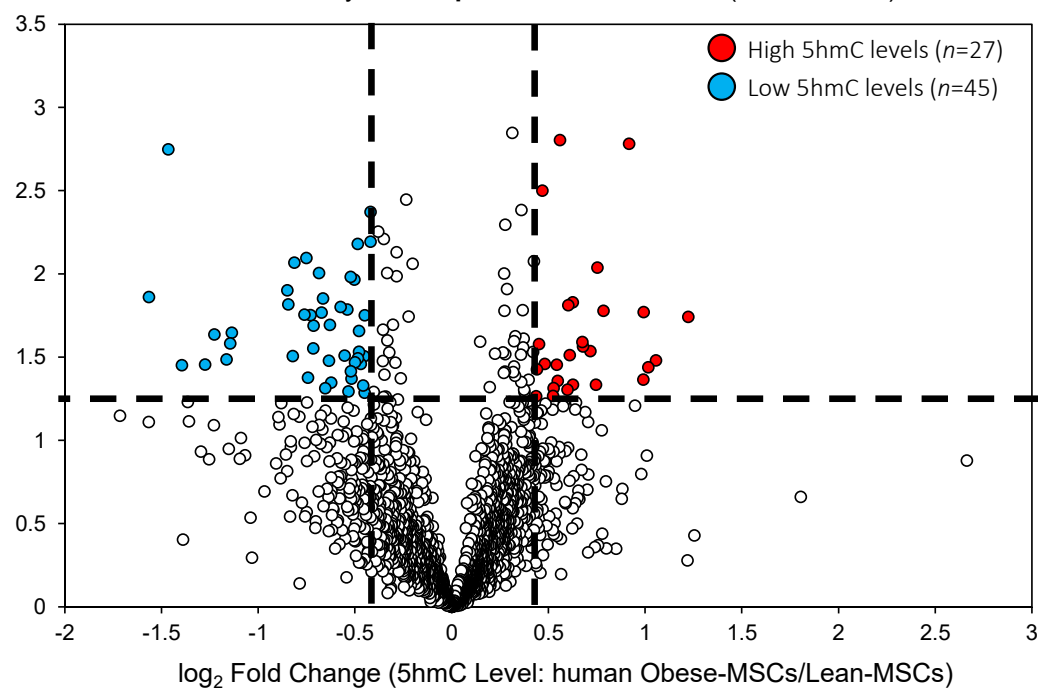

B

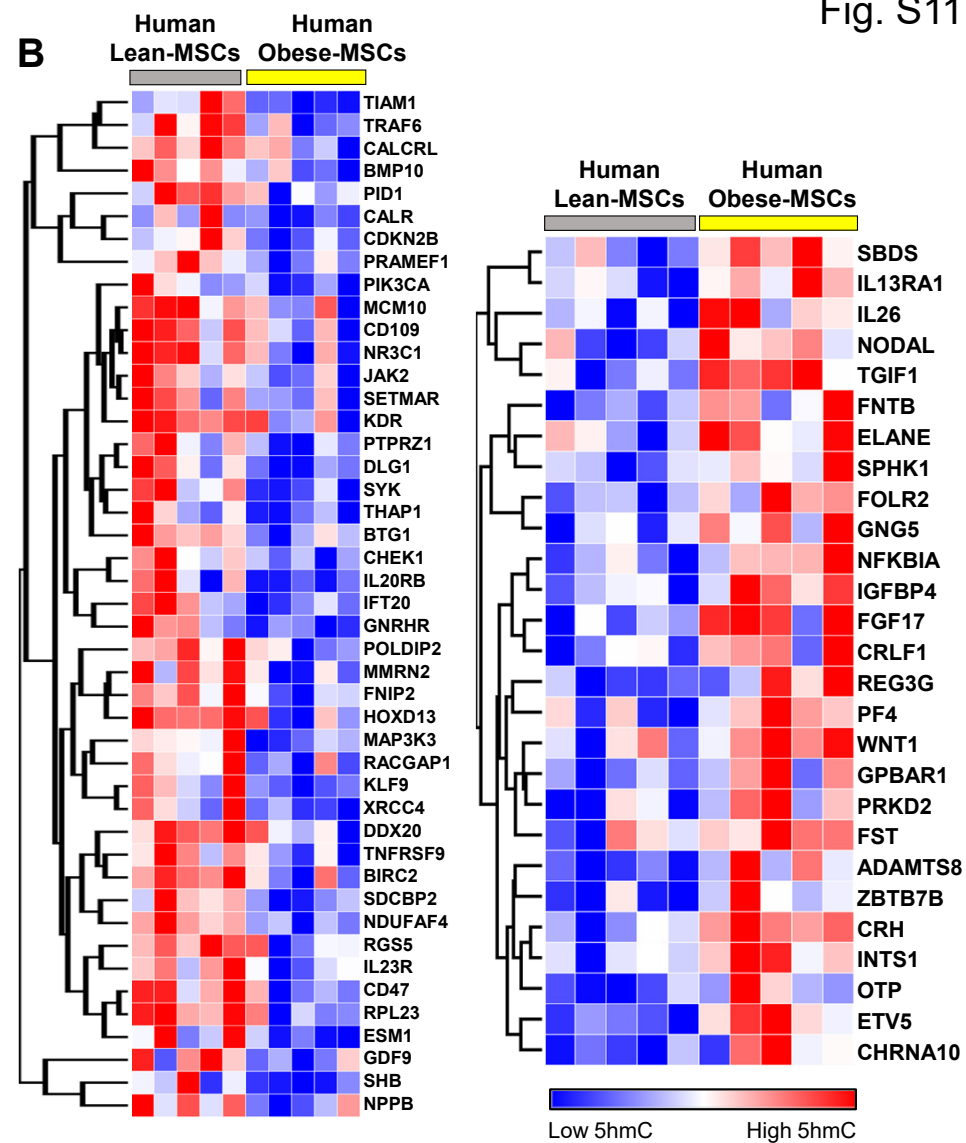

Supplement: Supplementary file 12 — Additional file 12: Figure S11. Obesity-driven dysregulation of 5hmC levels on genes related to cell population proliferation in human MSCs. A Volcano plot showing differential 5hmC levels in human Obese- versus Lean-MSCs for genes filtered by the GO term for cell population proliferation. For a given gene, differential 5hmC levels entail p-value ≤ 0.05 and fold change ≥ 1.4 or ≤ 0.7. Genes with high or low 5hmC levels in Obese-MSCs versus Lean-MSCs are represented with red or blue markers, respectively. B Heat maps of genes filtered by cell population proliferation showing lower or higher 5hmC levels in human Obese-MSCs versus human Lean-MSCs. [file 13287_2023_3372_MOESM12_ESM.pdf]
