# Supplementary material for: Obesity and dyslipidemia are associated with partially reversible modifications to DNA hydroxymethylation of apoptosis- and senescence-related genes in swine adipose-derived mesenchymal stem/stromal cells
Source: Stem Cell Res Ther. 2023 May 25;14:143. doi: 10.1186/s13287-023-03372-x (PMC10214739; doi:10.1186/s13287-023-03372-x)
Supplement: Supplementary file 6 — Additional file 6: Figure S5. Additional cell proliferation studies on swine Obese- and Lean-MSCs. Percentage cellular positivity for A PCNA or B Ki67, determined by immunofluorescent staining and fluorescence microscopy, did not differ in Obese- versus Lean-MSCs. PCNA: p-value = 0.66, Lean-MSCs: n = 6, Obese-MSCs: n = 5; Ki67: p-value = 0.89, n = 6 per group. C MTS assay showed decreased background-corrected absorbance at 490 nm in Obese-MSCs compared with Lean-MSCs, n = 6 per group; means of duplicate or triplicate measurements. *p-value = 0.0087 vs. Lean-MSCs. [file 13287_2023_3372_MOESM6_ESM.pdf]

Fig. S5

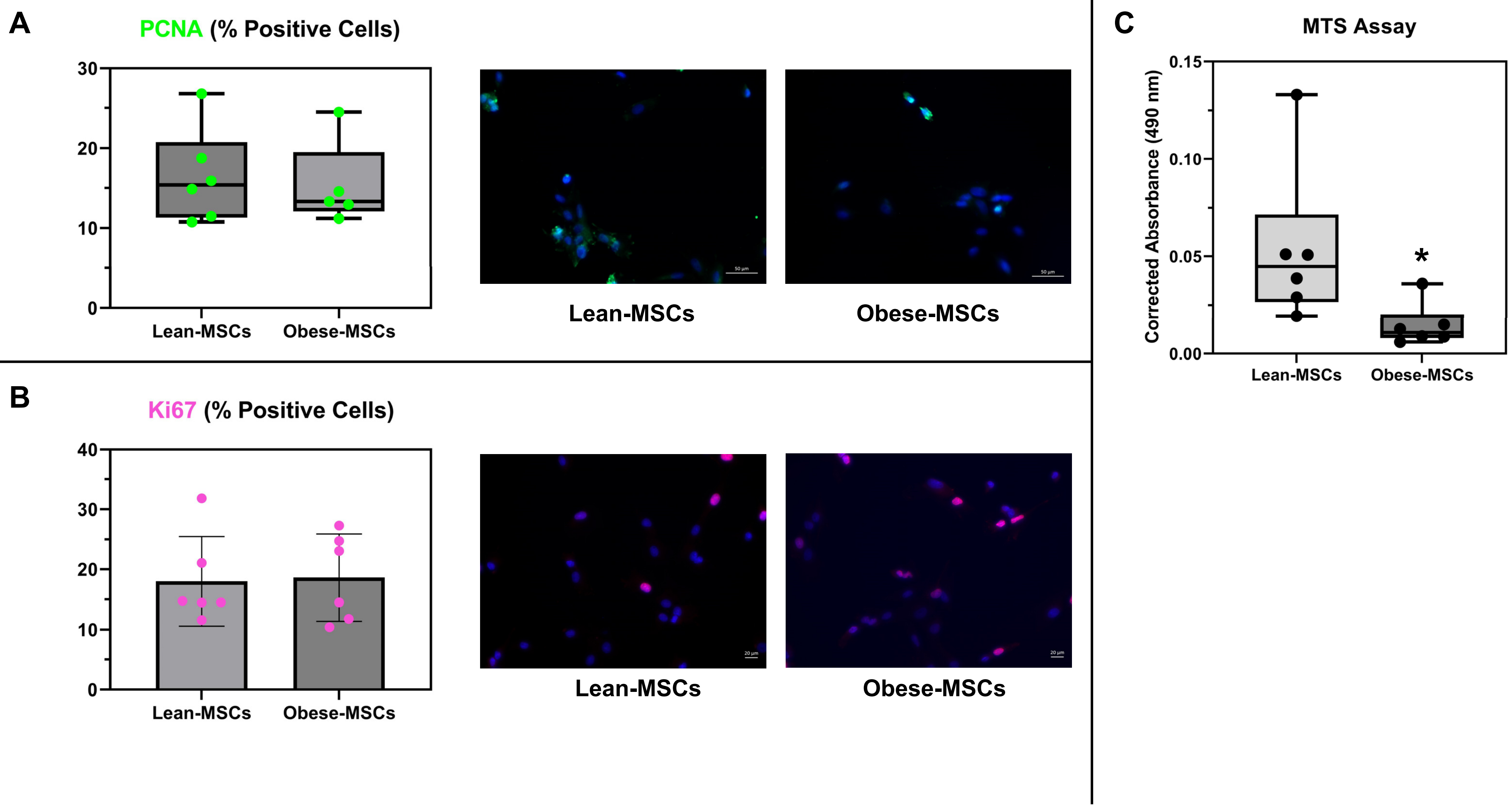

A
PCNA (% Positive Cells)
30
20
10
0
Lean-MSCs
Obese-MSCs
Lean-MSCs
Obese-MSCs
B
Ki67 (% Positive Cells)
40
30
20
10
0
Lean-MSCs
Obese-MSCs
Lean-MSCs
Obese-MSCs
C
MTS Assay
Corrected Absorbance (490 nm)
0.15
0.10
0.05
0.00
*
Lean-MSCs
Obese-MSCs
