# Supplementary material for: Obesity and dyslipidemia are associated with partially reversible modifications to DNA hydroxymethylation of apoptosis- and senescence-related genes in swine adipose-derived mesenchymal stem/stromal cells
Source: Stem Cell Res Ther. 2023 May 25;14:143. doi: 10.1186/s13287-023-03372-x (PMC10214739; doi:10.1186/s13287-023-03372-x)
Supplement: Supplementary file 7 — Additional file 7: Figure S6. Apoptosis/cell death assay of swine Obese- and Lean-MSCs. Representative flow cytometry scatterplots of MSC apoptosis/cell death tested using Annexin-V and Sytox for swine A Lean- and B Obese-MSCs. The red panel represents live cells, the orange panel represents dead cells, and the yellow panel represents apoptotic cells. [file 13287_2023_3372_MOESM7_ESM.pdf]

Fig. S6

A

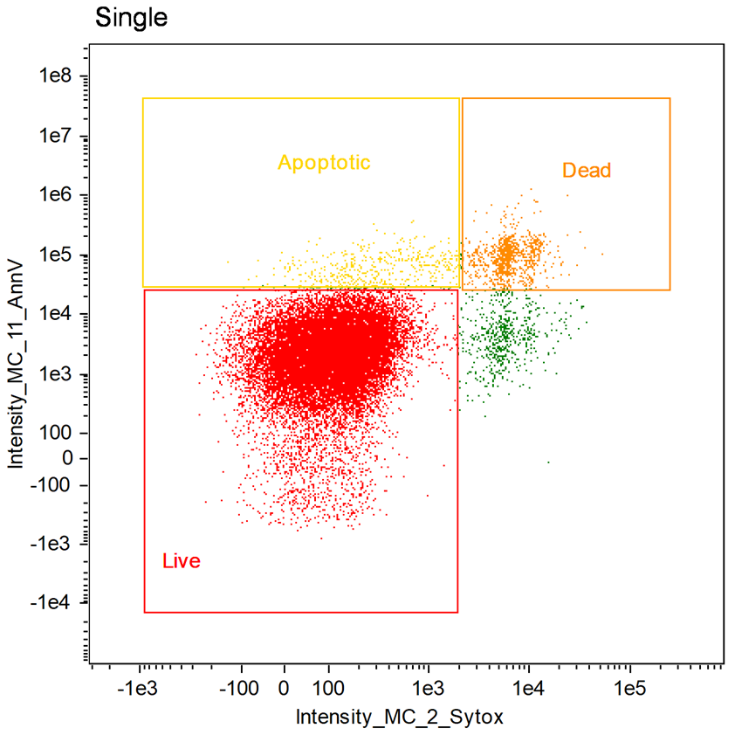

Intensity_MC_2_Sytox, Intensity_MC_11_AnnV

Population	Count	%Gated
Single & Focused	23139	100
Live & Single & Focused	21124	91.3
Apoptotic & Single & Focused	422	1.82
Dead & Single & Focused	972	4.2

B

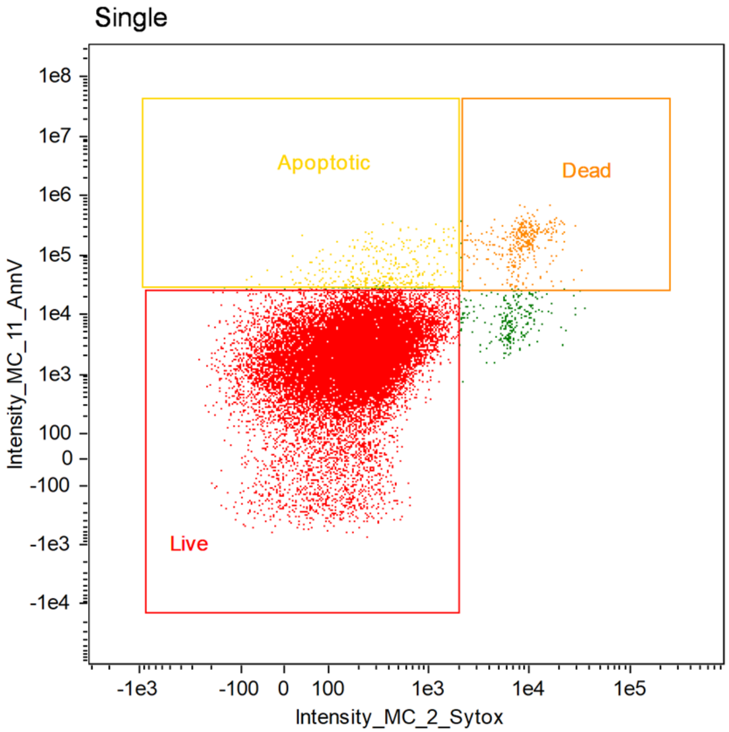

Intensity_MC_2_Sytox, Intensity_MC_11_AnnV

Population	Count	%Gated
Single & Focused	23328	100
Live & Single & Focused	22296	95.6
Apoptotic & Single & Focused	332	1.42
Dead & Single & Focused	358	1.53
