# Supplementary material for: Obesity and dyslipidemia are associated with partially reversible modifications to DNA hydroxymethylation of apoptosis- and senescence-related genes in swine adipose-derived mesenchymal stem/stromal cells
Source: Stem Cell Res Ther. 2023 May 25;14:143. doi: 10.1186/s13287-023-03372-x (PMC10214739; doi:10.1186/s13287-023-03372-x)
Supplement: Supplementary file 9 — Additional file 9: Figure S8. Fluorescence microscopy TUNEL images in swine MSCs treated with a pro-apoptotic agent. Apoptosis was evaluated by terminal deoxynucleotidyl transferase dUTP nick-end-labeling assay in swine Lean- and Obese-MSCs treated either with staurosporine in DMSO at 20 nM for 24 h or with DMSO only. Representative images of DAPI-stained nuclei, TUNEL-labeled nuclei, and the merged channels are shown. Obese-MSCs showed attenuated development of apoptosis in response to staurosporine. [file 13287_2023_3372_MOESM9_ESM.pdf]

Fig. S8

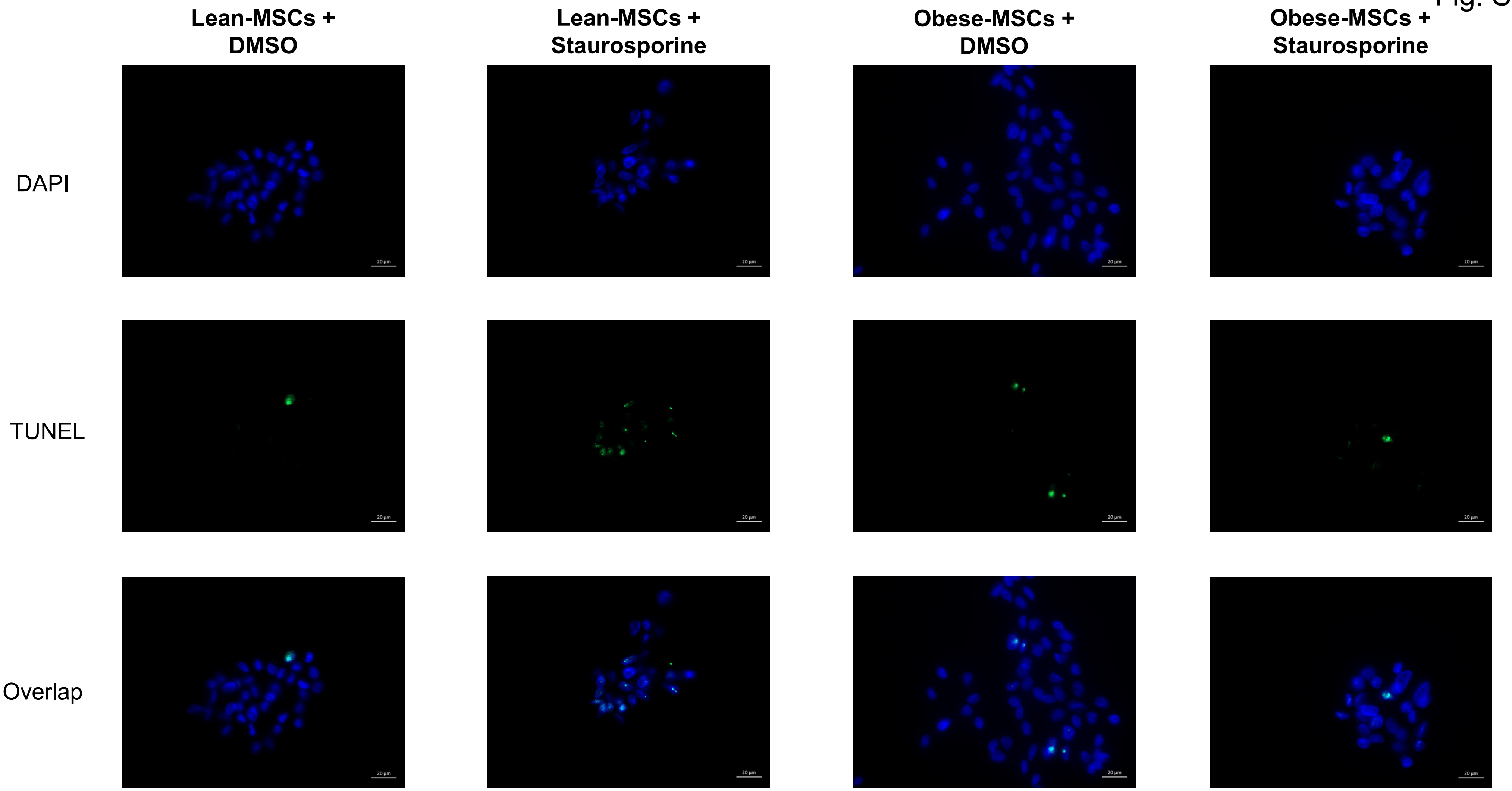

Lean-MSCs + DMSO
Lean-MSCs + Staurosporine
Obese-MSCs + DMSO
Obese-MSCs + Staurosporine
DAPI
TUNEL
Overlap
