# Supplementary material for: Obesity and dyslipidemia are associated with partially reversible modifications to DNA hydroxymethylation of apoptosis- and senescence-related genes in swine adipose-derived mesenchymal stem/stromal cells
Source: Stem Cell Res Ther. 2023 May 25;14:143. doi: 10.1186/s13287-023-03372-x (PMC10214739; doi:10.1186/s13287-023-03372-x)

Fig. S10

Human MSCs

A

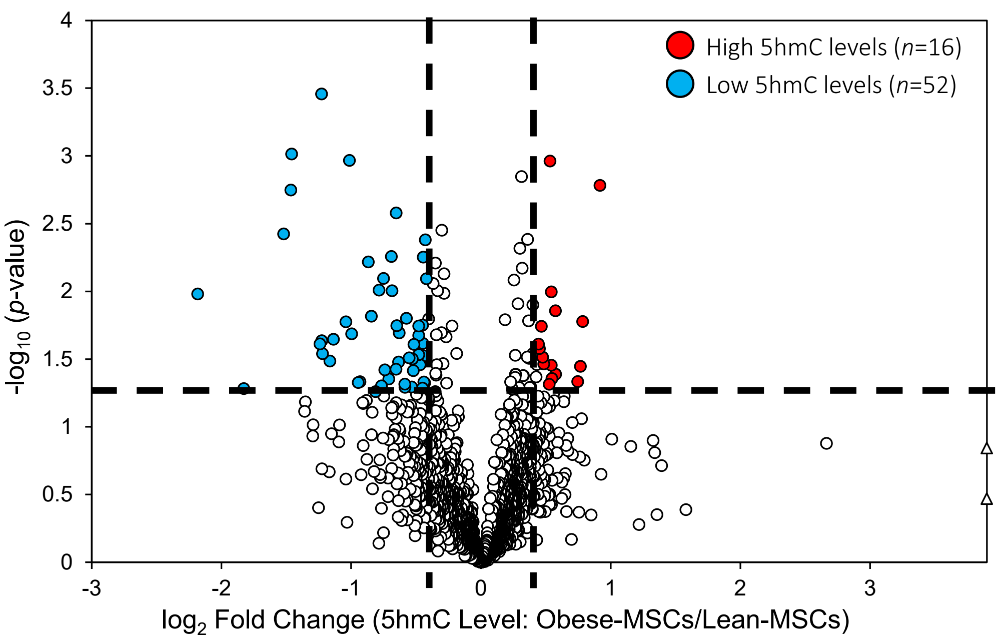

B

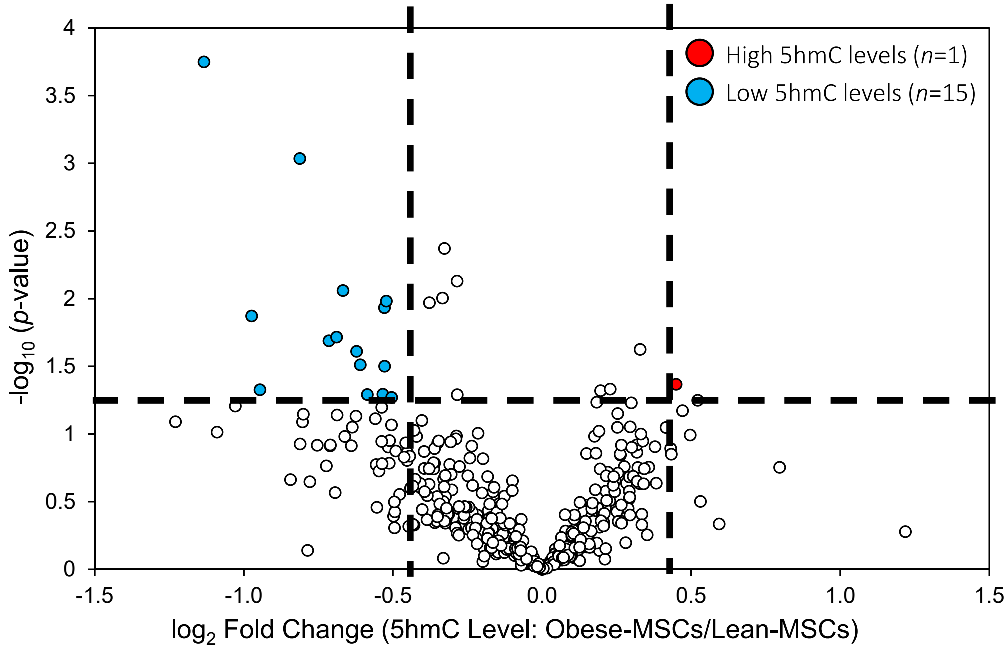

Supplement: Supplementary file 11 — Additional file 11: Figure S10. Obesity-driven dysregulation of 5hmC levels on genes related to apoptotic process and cellular senescence in human MSCs. Volcano plots showing differential 5hmC levels in human Obese- versus Lean-MSCs for genes filtered by A the GO term for apoptotic process or B the union of the GO term and REACTOME Superpath for cellular senescence. For a given gene, differential 5hmC levels entail p ≤ 0.05 and fold change ≥ 1.4 or ≤ 0.7. Genes with high or low 5hmC levels in Obese-MSCs versus Lean-MSCs are represented with red or blue markers, respectively. Genes with very high fold change in 5hmC levels of Obese- versus Lean-MSCs are presented as outliers with triangle markers. [file 13287_2023_3372_MOESM11_ESM.pdf]
